# Errors in the Laboratory X‑ray Absorption Near Edge Structure with Respect to Species Analysis in Slags Considering Leak Radiation

**DOI:** 10.1021/acs.analchem.6c02072

**Published:** 2026-09-30

**Authors:** Jessica Hiller, Joanna Kolny-Olesiak, Sven Hampel, Thomas Schirmer, Ursula E. A. Fittschen

**Affiliations:** † Institute of Inorganic and Analytical Chemistry, 26534 Clausthal University of Technology, 38678 Clausthal-Zellerfeld, Germany; ‡ Institute of Geotechnology and Mineral Resources, 26534 Clausthal University of Technology, 38678 Clausthal-Zellerfeld, Germany

## Abstract

The knowledge on the element species in slags is of great importance for, e.g., process control or recovery of valuable materials. X-ray absorption near-edge spectroscopy (XANES) is a suitable method, especially for slags, since it allows element speciation not only in crystalline but also in amorphous phases. The sample preparation as well as the evaluation via linear combination fitting of reference spectra are relevant influencing factors for the results. Errors can occur due to, e.g., improperly prepared samples, damping effects, and inappropriate reference materials. In this study, the origin of spectral damping was investigated with two lab-based spectrometers on a sample series of Fe_2_O_3_ prepared as pellets with different masses. Additionally, an electric arc furnace (EAF) slag served as a complex model sample to determine the influence of dampened spectra on the speciation analysis through linear combination fitting. Powder X-ray diffraction (PXRD), X-ray fluorescence spectrometry (XRF), electron probe microanalysis (EPMA), and inductively coupled plasma optical emission spectroscopy (ICP-OES) were employed as complementary and supporting characterization methods.

## Graphical abstract

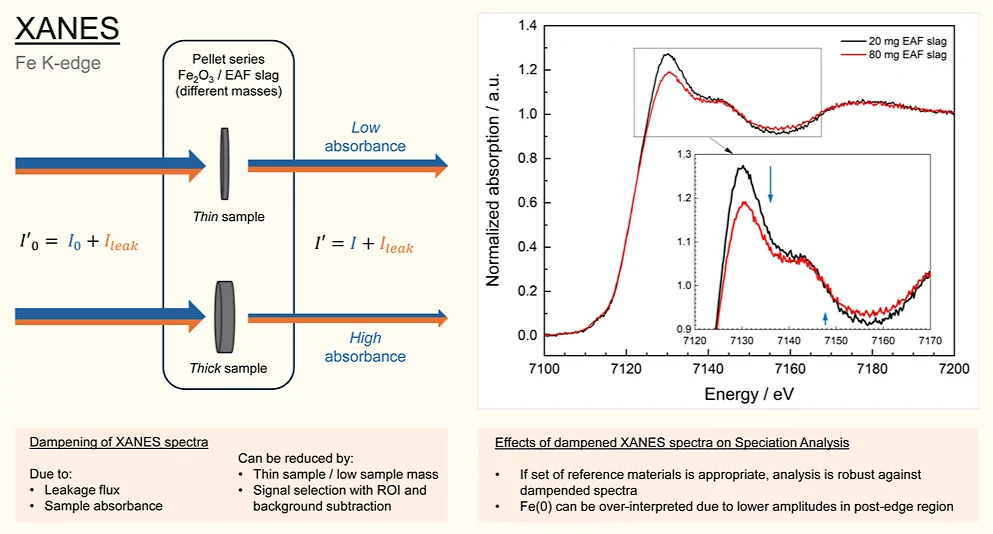

## Introduction

Solid-state species (e.g., oxidation state and nature of nearest neighbors) determination is of utmost importance in many fields like catalysis, nuclear research, geochemistry, and increasingly, the recovery of critical elements from waste streams. It can be accomplished *in situ* by photon-matter interaction, e.g., magic angle spinning nuclear magnetic resonance spectroscopy (MAS-NMR),^1^ high-energy resolution proton-induced X-ray emission (HR-PIXE),^2^ or several X-ray spectroscopy methods,^3^ such as X-ray photoelectron spectroscopy (XPS)^4^ and X-ray absorption fine structure (XAFS), including X-ray absorption near edge structure (XANES) as well as extended X-ray absorption fine structure (EXAFS).^5^ XAFS analyses can be applied to most elements and is bulk sensitive. Most XANES and EXAFS studies are performed at synchrotron facilities, making use of the high brilliance of this excellent source. However, XANES applications do not necessarily require this high brilliance, and X-ray tube based XAFS are sufficient for many samples with analyte concentrations higher than 1 to 2%. Laboratory XANES analysis has seen a renaissance in recent years.^6–8^ This is due to developments in instrumentation, detectors, sources, and optics and also driven by the need of modern condensed matter research to be able to study non-crystalline samples in the laboratory. Different designs of spectrometers are in use, e.g., Hámos type,^9–12^ Rowland circle,^13–15^ and off-Rowland circle.^16^ Each design comes with its unique set of advantages and sources of errors. Lately, a new configuration for X-ray absorption spectroscopy in combination with a standard laboratory X-ray powder diffractometer was reported by Gateshki et al.^17^

In recent years, the recovery of critical materials from waste streams like pyrometallurgical slags has become an area of intensive research.^18^ Along with increasing the supply risk of resources relevant for modern economies, e.g., rare earth elements, cobalt, and lithium, the necessity to recycle these materials has grown. In pyrometallurgical processes, ca. 12 million tons of steel slag are produced each year in Germany.^19^ They contain elements like tantalum (e.g., tin and electronic waste recycling slags), vanadium (e.g., some converter slags), lithium (e.g., from Li-ion battery recycling), and others in concentrations comparable to primary resources. The recovery and reuse of the slags require knowledge of the species of certain elements to (a) prevent environmental hazards, e.g., due to leaching of V^V^, and (b) harvest critical elements from enriched phases, e.g., LiMnO_2_ for the recovery of lithium.^20^ Goal (a) requires species determination in a dissolved state and relatively low limits of detection. Aqueous dissolved species determination at low concentrations can be achieved using, among others, chromatography separating the species in combination with a powerful detection tool like, e.g., mass spectrometry.^21–23^ Goal (b) requires solid-state species analysis. The oxidation state of redox-active metal ions in slags is highly important for the formation of recoverable compounds. The concentration of such metal ions is often quite high, e.g., iron ca. 20 wt % in electric arc furnace (EAF) slags and chromium and about 15% in ferrochromium slags. The lab-XANES spectra can yield highly valuable information on the metal species. The absorption spectra often exhibit unique features, like, e.g., pre-edge energy and intensity, main edge energy, whiteline, and oscillations. Linear combination fitting (LCF) is frequently used to estimate the species composition.^24,25^ In Figure 1, a typical normalized XAFS spectrum determined in transmission mode is shown. The edge is determined by the maximum of the first derivative. At approx. 50 eV above the edge, the spectrum can be divided into XANES and EXAFS regions. The XANES pre-edge and whiteline are distinct features resulting from the electronic structure of the core element. The EXAFS exhibits oscillation, which can be modeled from the distances and nature of next neighbors.^5^

**Figure 1:**
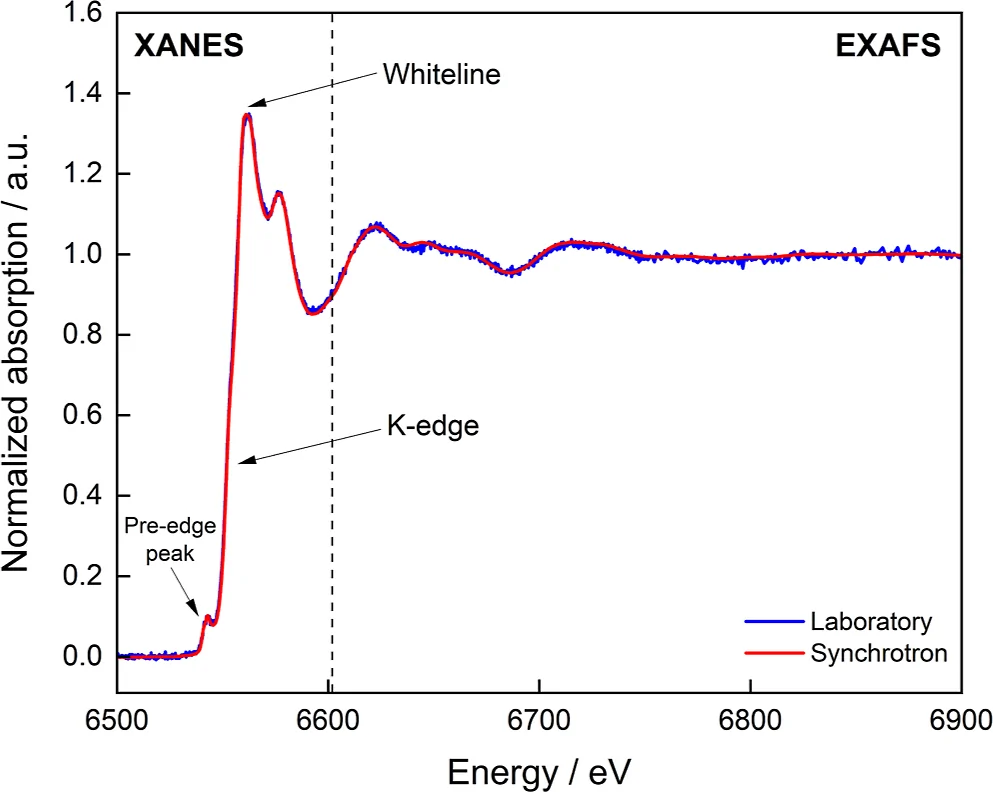
Exemplary XAFS for manganese­(IV) oxide measured as a pellet in the transmission mode with a laboratory setup (easyXES-100, blue line) and at the BAMline endstation at BESSY II (red line). Characteristic features are given with the pre-edge peak, the edge, and the whiteline. Experimental details are available in the Supporting Information (Tables S1 and S2).

### Errors in Absorption Measurements Due to Leak Radiation

Errors introduced by leak radiation are well known to absorption spectrometry in general. Depending on the spectrometry, it is sometimes also termed “parasitic radiation” or “stray radiation”. Leak radiation is the part of the primary radiation, which cannot be absorbed by the sample. The presence of leak radiation leads to a damping of the absorbance, especially for thick or highly concentrated samples. In AAS, this is observed in the curvature of the calibration curve at high concentrations.^26,27^ Different sources of leak contributions are illustrated in Figure 2 and examples from different absorption spectroscopies, i.e., atomic absorption spectrometry (AAS), ultraviolet and visible light absorption spectroscopy (UV-vis), and XAFS from the literature, are given in Table 1. Spectral leak radiation can occur from the parts of scattering background, as described by Hogness et al.^28^ in UVVis spectral photometry. This is illustrated in Figure 2A (for A–E, black: spectrum at the detector, green: absorption line, and orange: leak part). Fluorescence from the sample can contribute to the leak fraction in XAFS once the energy has surpassed the energy for photoelectron production (Figure 2B).^29^ Broadening of the emission line, e.g., of the hollow cathode lamp in AAS, results in a considerable part of leak radiation described by several authors^26,27^ and is illustrated in Figure 2C. It also illustrates the monochromator wing function in XAFS, which, however, can have a stronger tailing to the low energy side (Johann error, Figure 2D).^30^ Higher harmonics like those illustrated in Figure 2E have been addressed as a source of leak radiation in XAFS^31^ as well. Non-spectral contribution, like uneven atomic distribution of the atoms in the graphite furnace in GF-AAS, has been identified as the leak source by Gilmutdinov et al.^32^ This is illustrated in Figure 2F.

**Figure 2:**
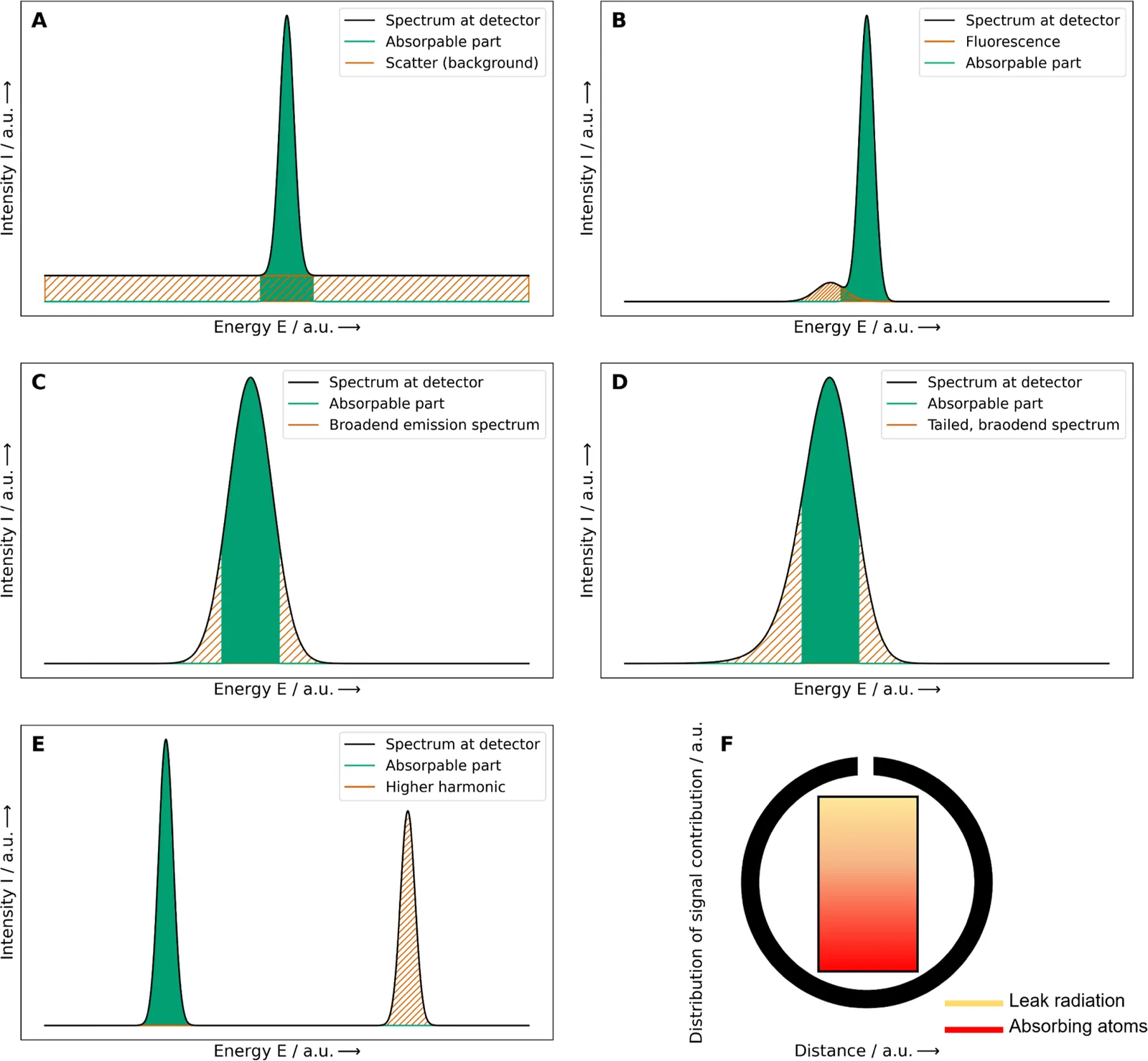
Illustration of leak flux origin. Spectral contributions are shown in A–E. Black, spectrum at the detector; green, absorption line; and orange, leak fraction. Leak fraction coming from scattering (A), emission from fluorescence (B), emission line broadening (C), asymmetric broadening (D), and higher harmonics (E). Contributions due to inhomogeneous distribution is illustrated in (F), where a cross section of a graphite tube together with the flux coming through the monochromator band path is shown, red is the uneven absorption, and yellow is the uneven leak fraction due to uneven atom distribution.

**Table 1:** Leak Radiation in Absorption Spectroscopy, Observation, and Discussion on Origins

method wave length	observation	description	leak sources discussed	Class Figure 2	citation
flame AAS 324.8 nm, 213.8 nm,285.2 nm	absorbance not keeping pace with concentration	absorption “diluted” by light which escapes absorption	broad emission line	C	Menzies^26^
			flame height zone of poor absorption	F	
flame AAS 324 nm, 328 nm	analytical curves are appreciably bent		HCL line profile Doppler broadening	C	Wagenaar, deGalan^27^
GF-AAS 225.9 nm, 283.3nm, 308.2 nm	curvature of the calibration curve		spectral line distribution of absorbing atoms	F	Gilmutindov^32^
UV-vis 272.5 nm K_2_CrO_4_	error in the absorption coefficient	scattered radiation partially absorbed	scattered radiation from a continuous source	A	Hogness^28^
EXAFS 8980 eV	amplitudes are significantly perturbed	radiation whichis not attenuated as greatly	harmonics	E	Stern, Kim^33^
			scatter	A	
			monochromator tail	D	
			non-uniform sample	F	
EXAFS 8980 eV	amplitudes decrease		fluorescence	B	Babanov^29^
			non-uniform sample	F	
XANES 17164 eV	whiteline reduction	estimate total leak using detector offset	harmonics	E	Mottram^34^
			scatter	A	
			monochromator tail	D	

Leak radiation and its effect on the calibration line curvature have been extensively studied in AAS (Table 1). Perhaps the most systematic and relevant contribution to this topic in XAFS is the work from Stern and Kim,^33^ who provided a theoretical description of the damping of the EXAFS amplitude and an experimental investigation of copper foils of varying thickness. Since the thickness effect is actually a mass attenuation effect (a function of both thickness and concentration), and with increasing use of XANES data, there is a strong need to characterize the influence of mass concentration in, e.g., pellet samples, particularly with respect to whiteline reduction in XANES. Mottram et al.^34^ observed sample thickness and leakage effects in an investigation on cerium and uranium XANES. Unfortunately, their compensation strategy is not entirely clear, and a systematic evaluation was not applied. Since instrument settings and data evaluation strategies can have a major impact on the results of analysis when using commonly applied LCF methods, it is crucial to study the magnitude of leak radiation, its influencing factors, their origin, and their influence on LCF results. Hence, in this study, we investigate the origin and contributing sources of this leak radiation and their effects on speciation analysis. We employ a systematic approach based on the preparation and measurement of pellet samples with increasing mass concentration. The pellet preparation allows the incorporation of powdered material and is suitable for various material types. Starting with pellets that contain Fe_2_O_3_ as a simple sample matrix, we are able to perform a systematic investigation of the leakage sources based on the recorded XANES spectra while simultaneously linking the impacts with the mass concentration. Subsequently, a series of pellets containing an iron-containing slag serves as a complex model system to examine the influence of sample mass concentration on the results of speciation analysis.

### Statistical and Systematic Errors in XAFS

The quality of the spectra can be described by noise and the spectral resolution of characteristic features. Statistical noise of the absorption mainly stems from the statistical errors of the primary *I*_0_ and the attenuated *I*. Rose and Shapiro^35^ have discussed statistical errors in XAFS in detail and have derived the optimal transmission (equivalent to the optimal thickness and optimal absorbance) of the specimen. Using error propagation, Rose and Shapiro determined the optimal transmission of a specimen to be 0.1. They also found that the optimal transmission increases because of leak radiation from 0.1 to 0.3. This means a shift of the optimum to thinner specimens with higher leak fraction (termed background by the authors). Furthermore, they considered the influence of a leak current, which is a fraction of the initial intensity of the beam *I′*_0_, which the sample cannot absorb. The background α is the fraction of the leak current *I*_leak_ relative to the absorbable radiation *I*_0_

$$\alpha =\frac{I_{leak}}{I_{0}}$$ 1

The measured intensities are *I′*_0_ the primary beam intensity and *I′* the attenuated intensity behind the absorbing sample. The true primary and the true attenuated intensities, which are also those supposed to be used in Beer-Lambert’s law, are *I*_0_ for the absorbable primary beam intensity and *I* for the attenuated beam of this fraction

2 $$I_{0}^{\prime }=I_{0}+I_{leak}$$

3 $$I^{\prime }=I+I_{leak}$$

The optimal sample would be the one with the lowest statistical error of the absorbance μ·*x*. The error depends on the intensities of *I*_0_, *I*_leak_ and *I*. The leak current and the primary beam intensity cannot be changed, so the only parameter which can be optimized is *I*. This is done by either optimizing the sample thickness or the mass per area concentration. The optimized parameters will then yield an optimal transmission of the sample. The dependence of the absorbance and the transmission *T* is given by Beer-Lambert’s law

$$I=I_{0}\cdot \exp\left(-\frac{\mu }{\rho }\cdot \rho \cdot x\right)=I_{0}\cdot \exp\left(-\frac{\mu }{\rho }\cdot \frac{m}{A}\right)=I_{0}\cdot \exp\left(-\mu_{lin}\cdot x\right)$$ 4

5 $$T=\frac{I}{I_{0}}$$

6 $$\mu_{lin}=-\frac{1}{x}\cdot \ln\left(T\right)$$

Using Beer-Lambert’s law, the product from density and thickness can be expressed as area density with the sample mass *m* and the area *A*. Note that rewriting eq 4 with the linear total absorption coefficient [cm^–1^] the thickness is introduced, again. Assuming that only the transmission is associated with an error (Δ indicating absolute standard errors), the relative error of the absorbance, *p* becomes

7 $$p=\left|\frac{\Delta \mu_{lin}}{\mu_{lin}}\right|=\left|\frac{\delta \mu_{lin}}{\delta T}\cdot \frac{\Delta T}{\mu_{lin}}\right|$$

With

8 $$\frac{\delta \mu_{lin}}{\delta T}=-\frac{1}{x\cdot T}$$

And substituting μ_lin_ from (eq 6) *p* becomes

9 $$p=\left|\frac{\Delta T}{T\cdot \ln\left(T\right)}\right|$$

With no leak current

10 $$\frac{\Delta T}{T}=\sqrt{{\left(\frac{\Delta I_{0}}{I_{0}}\right)}^{2}+{\left(\frac{\Delta I}{I}\right)}^{2}}$$

The leak current is included twice in the error propagation. The statistical error *p* is then modeled for different values of α.

The leak current not only adds to the statistical noise but also adds a component to the measured signal that cannot be absorbed. This systematic contribution of background leads to a damping of the fine structure. The damping of the absorbance is calculated using the formalism described by Stern and Kim^33^ for the amplitude reduction. The ratio of the measured *I′*_0_ and *I′* is then given as follows

11 $$\exp\left(\mu_{lin}^{\prime }\cdot x\right)=\frac{I_{0}^{\prime }}{I^{\prime }}=\frac{I_{0}+I_{leak}}{I_{0}\cdot \exp\left(\mu_{lin}\cdot x\right)+I_{leak}}$$

The reduction of the whiteline, or more accurately of the absorbance, is then given by the relation of the measured absorption over the true absorption

12 $$\frac{\mu_{0}^{\prime }}{\mu_{0}}=\frac{\ln\left(T^{\prime }\right)}{\ln\left(T_{true}\right)}$$

These errors limit the determination of different species, e.g., using linear combination fits.

Only for very thin samples (of low absorbance) is the damping in absorption spectroscopy negligible. The magnitude of the leak radiation, given as the fraction of the non-absorbable radiation *I*_leak_ over absorbable radiation *I*_0,true_, is often referred to as “true” primary radiation (eq 1).The leak radiation leads to a systematic reduction of the absorbance, which is stronger when the absorbance is high, e.g., at strong absorption bands like the whiteline in the XANES, reducing characteristic features. This phenomenon—in case the leak current is constant over the scanned energy range—is illustrated in Figure 3. The primary radiation *I′*_0_ (blue) consists of absorbable radiation and leak part *I*_leak_ of about 110 counts (grey). The true attenuation *I*_true_ (red) is dampened whenever the attenuation exceeds the 110 counts, resulting in the observed attenuated *I′* (yellow).

**Figure 3:**
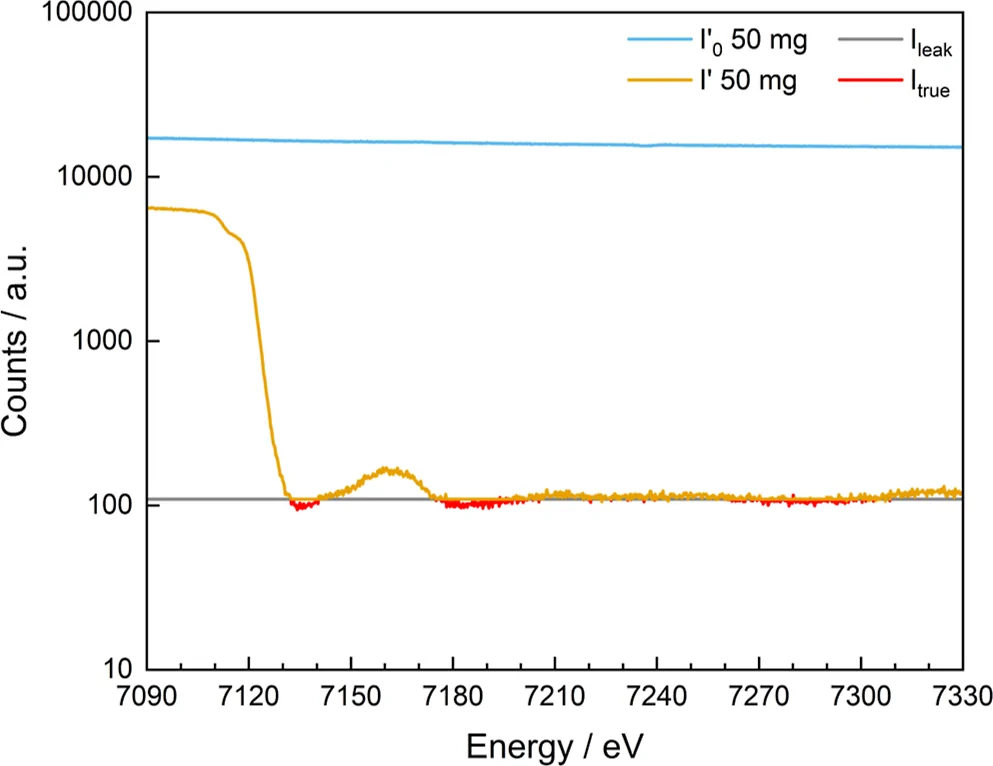
Illustration of the effect of the leak flux on the observed attenuated. The observed primary radiation *I′*_0_ (blue) has a leak part *I*_leak_ of about 110 counts (grey), which leads to a dampened observed attenuated *I′* (yellow) compared to the true attenuation *I*_true_ (red).

The higher the leak fraction, the higher the expected damping for samples with equal thickness. It can be modeled as a function of the leak fraction and the sample absorbance (thickness) using the Beer–Lamberts law. The reduction of the observed absorbance modeled for different fractions of leakage radiation is part of our investigation. Stern and Kim^33^ have derived the dependence of the EXAFS amplitude reduction from the leak fraction and studied the phenomenon by using Cu foils of different thicknesses.

Accordingly, they have termed the damping the thickness effect, although it actually depends on the absorbance. In conclusion, the sample needs to be as thin as possible to minimize systematic errors from leakage radiation, although there is an optimal thickness regarding the noise.

### Sources of Leak Flux in XAFS

At high leak fractions, damping occurs already with ultrathin samples, which can render an acceptable sample preparation impossible. The elimination of leakage requires identification of its most important sources. The origin of leak photons in XAFS has been discussed in the literature.^29,31,33,36,37^ It was suggested that scattered radiation not passing the sample (Figure 2A), higher-order harmonics (Figure 2E), void fraction of the specimen (Figure 2F), fluorescence (Figure 2B), and the monochromator energy wing contribute to it (Figure 2C,D). Different measures were suggested and applied for minimizing the contributions to leak radiation listed above, i.e., proper collimation of the detector to prevent scatter radiation and the preparation of finely powdered samples to prevent voids and particle size effects.^33,38^ Mottram et al.^34^ used a detector offset to determine the leak radiation; however, their rationale is not clear, and the spectra were neither shown nor described.

The fraction of stray radiation from the different sources described above can vary across the energy range. This is particularly evident for the low-energy tail of a monochromator’s emission, which is significant at low angles of Johann crystals (Johann error).^30^ The contributions from fluorescence or scattering may also vary with energy, but their effect on the spectrum remains unclear. A mosaic crystal monochromator contributes a symmetric wing function to the stray radiation fraction, though the implications of this on the spectrum have not been isolated. The contribution from microstructural voids is expected to be largely constant across the scanned energy range; however, it may vary slightly due to differences in scattering cross sections.

Here, we study the influence of the mass concentration on the quality of Fe K-edge XANES spectra obtained from two laboratory-based X-ray absorption spectrometers. We use the observed statistical noise and the spectra damping to estimate each instrument and data processing set leak fraction. We evaluated the source of the stray radiation by modeling a constant leak fraction and a symmetric monochromator function. The results show that a constant leak fraction fits the data from the two instruments using Si(220) and Ge(400) crystals very well. The influence of different data processing procedures that represent specific contributions of leakage sources is investigated by considering different regions of interest of the energy-dispersive spectrum (available for one of the instruments). We use the first-order reflection with and without background subtraction and the entire spectrum. Subsequent to the evaluation of the leak fraction of the instrumentation, an actual EAF slag was studied with respect to the speciation of iron. The influence of sample absorbance on the errors in the linear combination fit is evaluated, and confidence intervals are determined.

## Experimental Section

### Element Determination in the Slag Material

#### X-ray Fluorescence Spectrometry

The XRF measurements were performed on pressed pellets by using the wavelength dispersive XRF spectrometer Axios (Malvern Panalytical, Worcestershire, United Kingdom). The pellets were composed of a binary mixture of wax and sample in a ratio of 1:5 (w/w) and were pressed at approx. 13 t with a laboratory press (HERZOG Maschinenfabrik GmbH & Co. KG, Osnabrück, Germany).

#### Electron Probe Microanalysis

Elemental concentrations in single grains of an EAF slag thin section were obtained using the electron probe microanalyzer SX FIVE FE (CAMECA SAS, Gennevilliers Cedex, France). The device is equipped with five WDX spectrometers and a set of four selectable crystals each. Calibration of the system was done on the basis of reference materials provided by P&H Developments Ltd. (Glossop, Derbyshire, United Kingdom) and Astimex Standards Ltd. (Toronto, Ontario, Canada). The measurements of the slag sample were performed at 15 kV, with a beam diameter of less than 1 μm (100–600 nm, Schottky-type^39^), and were accompanied by the generation of backscattered electron images with atomic number contrast (BSE­(Z)). For evaluating the measured X-ray emission intensities, the X-PHI-Model^40^ was used. Mineralogical phases were reconstructed from the relative element concentrations.

#### Wet-Chemical Digestion

Prior to the determination of the iron content by inductively coupled plasma optical emission spectroscopy (ICP-OES), the transformation of the solid slag material into a residue-free solution was necessary. Therefore, an aliquot of the pre-ground slag (*d*_90_ < 100 μm), i.e., 200 mg, was digested by performing a two-step procedure in a lab microwave system (Discover SP-D 80, CEM corporation, USA) with a mixture comprising 8 mL of 65% (m/v) nitric acid (p.a.; Carl Roth GmbH + Co. KG, Karlsruhe, Germany), 5 mL of 85% (m/v) *ortho*-phosphoric acid (p.a.; Merck KGaA, Darmstadt, Germany), and 1 mL of 40% (m/v) hydrofluoric acid (p.a.; Merck KGaA, Darmstadt, Germany) followed by a treatment with 6.5 mL of a saturated aqueous solution of orthoboric acid (p.a.; Merck KGaA, Darmstadt, Germany). Details about the digestion procedure can be found in the Supporting Information. The resulting digestion solution was light-green in color and did not contain any visible residues. Finally, the solution was transferred into a 100 mL volumetric flask and diluted with ultra-pure water (*ca*. 0.055 μS·cm^‑1^, Purelab Flex 4, ELGA Lab Water, High Wycombe, United Kingdom) to a final volume of 100 mL. In total, three slag samples as well as three blank samples (digestions without slag material) were prepared.

#### Inductively Coupled Plasma Optical Emission Spectrometry

Aliquots of all digested sample, and blank solutions were further diluted with the acid mixture used for the digestion and ultrapure water (dilution factor = 200). Calibration standards for ICP-OES measurements were prepared from certified single-element standard solutions (1 g·L^–1^; Carl Roth GmbH + Co. KG, Karlsruhe, Germany). Appropriate amounts of the acids employed in the digestions (except hydrofluoric acid) were added to adjust the standards to the acid concentrations in the diluted sample solutions. The calibration range covered element concentrations from 0 mg·L^–1^ to 10 mg·L^–1^. Measurements were performed with the ICP-OES instrument Varian Vista MPX (Varian Inc., Palo Alto, USA). The device was equipped with an autosampler, an echelle polychromator, a CCD array detector, a 40 MHz RF generator, and HF-resistant components. Argon 4.6 was used as plasma gas, a flow rate of 15 L·min^–1^ for feeding the torch was employed, and the optical system had a radial view on the plasma at a height of 12 mm. The iron emission signals at the wavelength 238.204 nm was chosen for analytical purposes, as this is the most sensitive emission line for iron. More detailed information about the sample preparation prior to the ICP-OES measurements and the measurements itself can be found in the Supporting Information (Table S3).

### Determination of Mineralogical Phases

#### Powder X-ray Diffraction

The EAF slag was prepared as a back-loaded specimen and measured with the diffractometer X-Pert Pro (Malvern Panalytical, Worcestershire, United Kingdom). The device is equipped with a Co anode-bearing X-ray tube (operated at 40 kV, 40 mA) and with the multiple strip X-ray detector PIXcel1D (Malvern Panalytical, Worcestershire, United Kingdom). Precision of the angular positions of the diffraction signals was checked on a regular basis using a standard silicon specimen. Measurements on the slag specimen were performed with a scan range of 5–100° 2θ, a step size of 0.013° 2θ, and a measurement time of 58 s per step. The recorded diffractogram was evaluated and interpreted based on the pdf-2 ICCD XRD database.^41^

### Analysis of element species

#### Sample Preparation

The Fe_2_O_3_ pellets were prepared using a polyethylene (PE) binder (Uvasol, Merck KGaA, Darmstadt, Germany) and targeting different area thicknesses of Fe (g·cm^‑2^). A constant PE mass of 80 mg was chosen while the mass of Fe_2_O_3_ was varied from 5 mg and 10 to 100 mg in 10 mg steps. The Fe_2_O_3_ and PE were finely mixed in an agate mortar and pressed into pellets of 16 mm diameter for 2 min at a pressure of 10 t (MP250, Maassen GmbH, Möglingen, Germany). Afterwards, the pellets were covered between Ultralene foil (SPEX SamplePrep, Metuchen, NJ, USA) and mounted into a 3D-printed pellet holder that fitted into the sample wheel of the XAFS spectrometer. *I*_0_ measurements were performed with a blank pellet consisting of 80 mg PE prepared in the same manner. Exemplary images of the pellets can be found in the Supporting Information (Figure S1).

The slag sample was an EAF slag, which was ground beforehand (*d*_90_ < 100 μm). Its Fe concentration was determined to be 312 ± 1 mg·g^–1^. Pellets of 8 mg as well as 20 mg to 80 mg in 10 mg steps were prepared with 80 mg PE as described above. References for the linear combination fit were prepared as described in Schirmer et al.^24^ 23 mg Olivine (mineralogical collection, TU Clausthal), with a major compound of Fe_2_[SiO_4_], 10 mg of Fe_2_O_3_ (99.9%, Thermo Fischer, Kandel, Germany), 10 mg of Fe_3_O_4_ (95%, Sigma-Aldrich, St. Louis, MO, USA), and 23 mg of iron chromite (>95%, Kremer Pigmente GmbH & Co. KG, Aichstetten, Germany) pressed into pellets as well as a Fe-metal foil (Exafs Materials, Danville, CA, USA) as the free-standing film were used. The film served also for energy calibration of the spectrometers. In total, three pellets were prepared with the slag and with each reference compound in order to compensate for inhomogeneities and to calculate the confidence intervals for the contents of the species determined in the slag.

#### XAFS Instruments and XANES Measurements

The EasyXES100 spectrometer (EasyXAFS, Renton, WA, USA) is equipped with a VF-80JM X-ray tube with the W/Pd anode operated at 3 mA/25 kV and a VITUS H80 80 mm^2^ SDD (Ketek, Munich, Germany). The energy resolution of the instrument is typically 0.5–1.5 eV at 85° Bragg angle and energies from 5000 to 12000 eV. The Fe K-edge was measured using a Si(220) SBCA. For the Fe_2_O_3_ pellet series, the energy scan was performed from 6912 to 7312 eV with a step size of ΔE = 0.25 eV and 1 s dwell time, resulting in 33 min per single scan. Ten repetitive measurements for *I* and *I*_0_ were carried out. The quantitative investigation of the slag regarding its iron species required different scan parameters. Here, the pellets of the reference compounds were scanned from 6930 to 7400 eV divided into three energy zones with different settings (Table S4). Since the EAF slag contained less iron than the reference compounds, the number of measurement repetitions was increased to 20 in order to improve the signal statistics. For each pellet, the respective *I* and *I*_0_ were merged. In the following, we will refer to this instrument as the EDX-based spectrometer, as the EDX features allow us to reduce or increase the leak fraction.

The QuantumLeap 2000 spectrometer (Sigray, Concorde, CA, USA) is equipped with a Dectris Eiger2R 500 K Si detector featuring 1030 × 514 pixels with a size of 75 × 75 μm. The X-ray tube consists of metal micro-structures (W, Mo) embedded in diamond powered at 20 kV/10 mA (200 W). The Fe K-edge spectra were recorded using the Mo target and a cubic crystal analyzer (CCA, Johansson-type) Ge­(220,400) crystal. They are located on a Rowland circle of 0.3 m diameter along further available crystals, namely Si­(220, 400) and highly ordered pyrolytic graphite (HOPG) flat optics. The instrument operates at energies from 4–14.8 keV. The energy scan settings are given in Table S4. In the following, we will refer to this instrument as the 2D-detector spectrometer, as this detector allows a semi-simultaneous spectra collection; however, it has less flexibility in removing artefacts from the spectrum.

The *I*_0_ data was obtained from PE blank pellets prior to the sample measurement. The absorbance at 7100 eV was used as background and subtracted from the spectra. For the normalization to the pre-edge, the absorbance was normalized to the one obtained at around 7112 eV and averaged for noisy data. The calculation of whiteline reduction μ′ over μ was done using the pre-edge normalized absorption at 7141 eV as μ′. Since the damping of the normalized absorption for the 5 mg sample is unknown, the data were analyzed using assumed whiteline reductions from 0% to 100%. For each assumption, the true μ was simulated based on the observed whiteline reduction in the normalized absorption of the 5 mg sample. These simulations were used to calculate reduction factors for the higher-concentration samples. The resulting data were compared to theoretical models, and the best fit was determined by minimizing the sum of squared deviations. The sum of squared deviations simulated from the EDX-detector spectrometer ROI data for the different whiteline reduction at 5 mg over the leak fraction are discussed and visualized in the section “Leak radiation sources”. The optimal fit provided the estimated leak fraction and the best damping value for the 5 mg sample. The EDX-detector spectrometer (ROI and FIT mode) yielded absorption factors of 0.989 and 0.997, respectively, while the 2D-detector spectrometer best fit with 0.813. The μ′ over μ obtained in this way was then plotted over the calculated absorbance μ·*x* at 7141 eV, considering only the Fe–K absorption with μ over ρ obtained from the McMaster et al. tables^42^ (403.66 cm^2^·g^–1^), the weight fraction of Fe, and the area (2.01 cm^2^) of the pressed pellet according to eq 4. The whiteline reduction for different leak fractions was modeled according to eqs 11 and 12.

### Simulation

To investigate the influence of the monochromator function of an X-ray absorption spectrometer on the spectra of samples with different thicknesses, a simulation approach using Python (PyCharm IDE, Python 3.10) was employed. Reference XANES spectrum of Fe_2_O_3_, obtained from a synchrotron database (XASLIB^43^), served as the basis for our calculations. This reference spectrum was utilized to derive the “true” transmission values for the sample. The thickness corresponding to this “true” transmission was arbitrarily designated as “one layer” of the sample. To explore the impact of different sample thicknesses, the transmission for thicker samples ranging from 2 to 10 layers was calculated. We simulated the monochromator function, which represents the spectral response of the spectrometer, using window functions. These window functions consisted of a triangular base with variable widths, simulating different lengths of the window tail, and a sharper triangular peak, which remained constant throughout the calculations. The transmission values obtained for different sample thicknesses were convoluted with the window function, thereby simulating the influence of the monochromator function on the damping of the whiteline in the spectra. Using *I*_0_ intensity from lab-based measurements (QuantumLeap 2000), a simulated lab-based *I* was obtained from the transmission. Consequently, the absorption corresponding to the convoluted transmission values was obtained. In order to facilitate the comparison of different spectra, the resulting absorption values were normalized by dividing them by the number of layers. Normalization processes were done in Larch.^44^ In this subsequent simulation, our objective was to explore the impact of leakage currents of varying intensities on the absorption spectra of samples with different thicknesses. The initial steps of the simulation remain the same as in the previous calculation. The mean value of the obtained intensity *I* was determined, and a certain percentage of this value was added to the calculated intensity *I*. This addition simulated the presence of leakage current, introducing variations in the intensity measurements while remaining the same *I*_0_ throughout all simulations. Using the modified intensity values (*I* with added leakage current) and the original *I*_0_ intensity, the absorption was calculated. Finally, the resulting absorption values were normalized by dividing them by the number of layers. This normalization facilitated the comparison of different spectra, accounting for variations in sample thickness.

## Results and Discussion

### Evaluation of Leak Flux (Alpha Factor)

The Fe K-edge XANES spectra of 11 specimens with Fe_2_O_3_ masses from 5 mg to 100 mg were obtained on two spectrometers to determine the fraction of the leak flux α of the two instruments. The 2D-detector spectra show a noticeable reduction in the whiteline intensity between the 5 mg and 10 mg samples (Figure 4a). In contrast, the spectra recorded with the EDX-based instrument only reveal a clear damping of the whiteline starting around 20 mg Fe_2_O_3_ (Figure 4b). To visualize the damping of the near-edge fine structure, the spectra were normalized on the pre-edge peak at *ca*. 7112 eV. The original non-normalized spectra are found in the Supporting Information (Figure S2).

**Figure 4:**
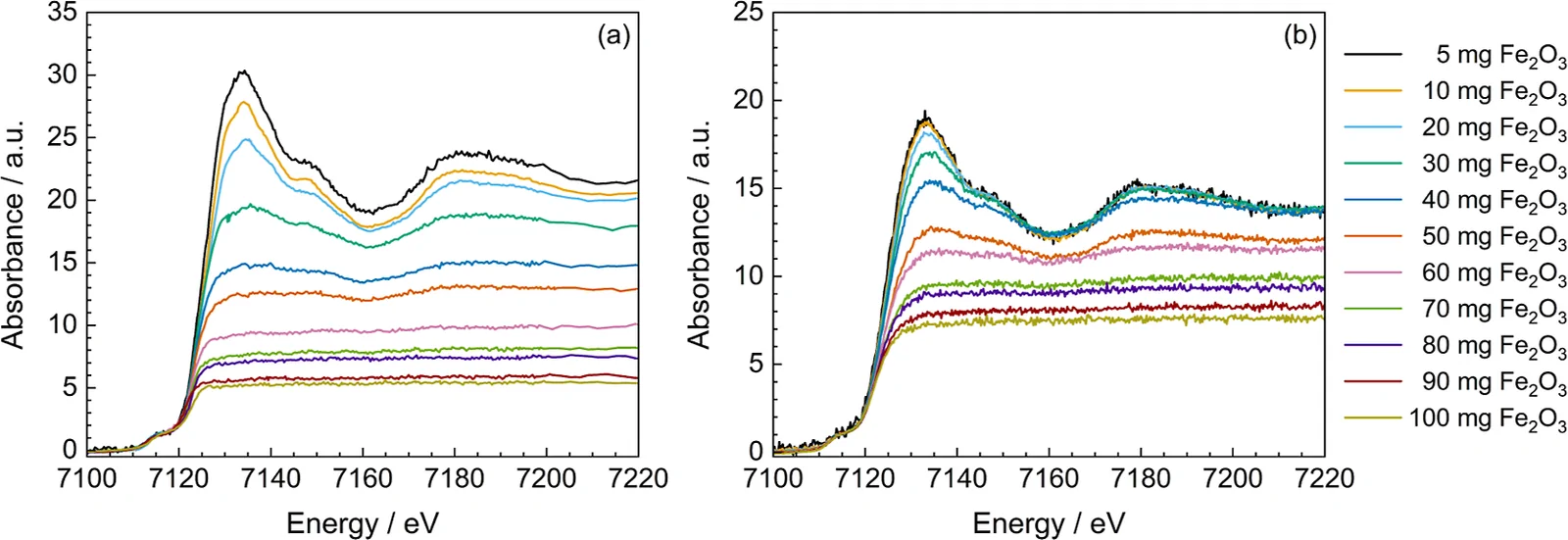
Fe K-edge absorption spectra normalized to the pre-edge (a) obtained from the 2D-detector instrument and (b) obtained from the EDX-based instrument.

Even though the EDX-based spectra in Figure 4b show low damping for the lower masses, the absolute absorbance is lower compared to calculated values (McMaster et al.^42^). Only the absorbance of the 10 mg sample matches the theoretical values, and the absorbance of the 5 mg sample is considerably lower than the theory, although the damping is lowest. Some experimental and theoretical absorption values at an energy of 7141 eV are given in Table 2. The lower absorbance of the 5 mg sample is very likely due to the inhomogeneity of the specimen, which is more pronounced because of the low amount of the Fe_2_O_3_ powder. This inhomogeneity of the 5 mg sample is clearly visible in the photo shown in Figure S1 in the Supporting Information. The lower homogeneity obviously does not lead to a significant damping, as can be seen in Figure 4, the 5 mg sample clearly shows the least dampened spectrum. The influence of the inhomogeneity as well as the assumption on the whiteline reduction of the lowest (5 mg) sample on the alpha factor estimate is discussed in the next section.

**Table 2:** Absorbance Measured and Calculated at 7141 eV for Fe_2_O_3_ Masses from 5 to 50 mg Analyzed with the EDX-Based Spectrometer

mass Fe_2_O_3_/mg	experimental—ln (*T*_FeK_)	calculated^a^ $\frac{\mu \cdot m}{\rho \cdot A}$
5	0.30	0.61
10	1.17	1.22
20	2.08	2.44
30	2.98	3.66
40	3.58	4.88
50	4.03	6.10

a Data from McMaster et al.^42^

The experimentally obtained noise of the spectra is the lowest for the 20 mg samples. The optimal transmission with respect to statistical noise is below 0.2 for specimens measured at the EDX-based spectrometer and above 0.2 for specimens measured at the 2D detector instrument. The relative noise *p* (eq 9) is plotted over the transmission (eq 5) in Figure 5.

**Figure 5:**
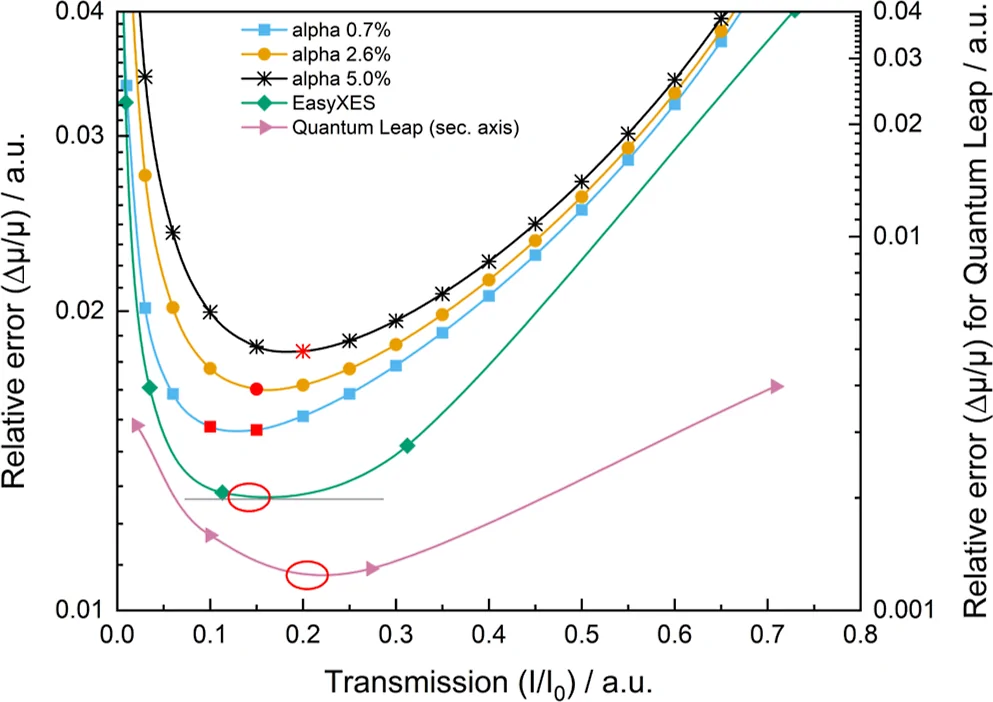
Relative noise calculated for different leak fractions and experimentally determined values.

### Evaluation of the True Absorbance of a Data Set

To estimate the leak fraction of each setting, the whiteline reduction of the 5 mg sample was obtained by simulating fitting errors for all whiteline reductions and determining the leak fraction (alpha) with the best fit. The results for the EDX-based spectrometer and the standard ROI fitting method are shown in Figure 6, along with the best fit alpha and corresponding the best fit whiteline reduction at 5 mg. In Table 3, the results for simulations of all instrument and setting data are shown, i.e., the best fitting alpha, the whiteline reduction at 5 mg and the deviation. The simulation suggests that a 1.2% alpha factor provides the best fit for the ROI fit of the EDX-based instrument, a 0.7% alpha factor fits best the background-fitted EDX spectra and a 16.6% alpha factor best fits the 2D-instrument (Figures S3 and S4). Using the entire EDX spectrum did not yield a clear error minimum but instead showed a gradual decrease in deviation with increasing alpha factor (Figure S5). The absence of a minimum suggests that the model may be under-constrained for the full-spectrum fit. From the ROI counts and the total counts of the EDX spectra in the whiteline region, a leak fraction of approx. 186% is derived, which would predict a whiteline reduction of 0.279. The results are also sensitive to the chosen reference database, i.e., McMaster et al. or Schoonjans et al.^42,45^ In conclusion, the sensitivity test shows that the data set can be used to obtain a good estimate of the whiteline reduction of the 5 mg sample. The model remains stable unless we use unrealistic values, then deviations increase significantly (Figure S6). This suggests the model is primarily guided by the trend established from all samples (5 mg to 80 mg). While the 5 mg sample has limitations, the model’s consistency across Fe_2_O_3_ concentrations supports the alpha factor estimate. Reducing the true absorbance of the 5 mg sample to lower values has no noticeable effect on the fit (Figure S7). Using the measured absorbance value (0.30) for the 5 mg sample, the deviation changes only slightly, meaning this assumption does not significantly affect the results.

**Figure 6:**
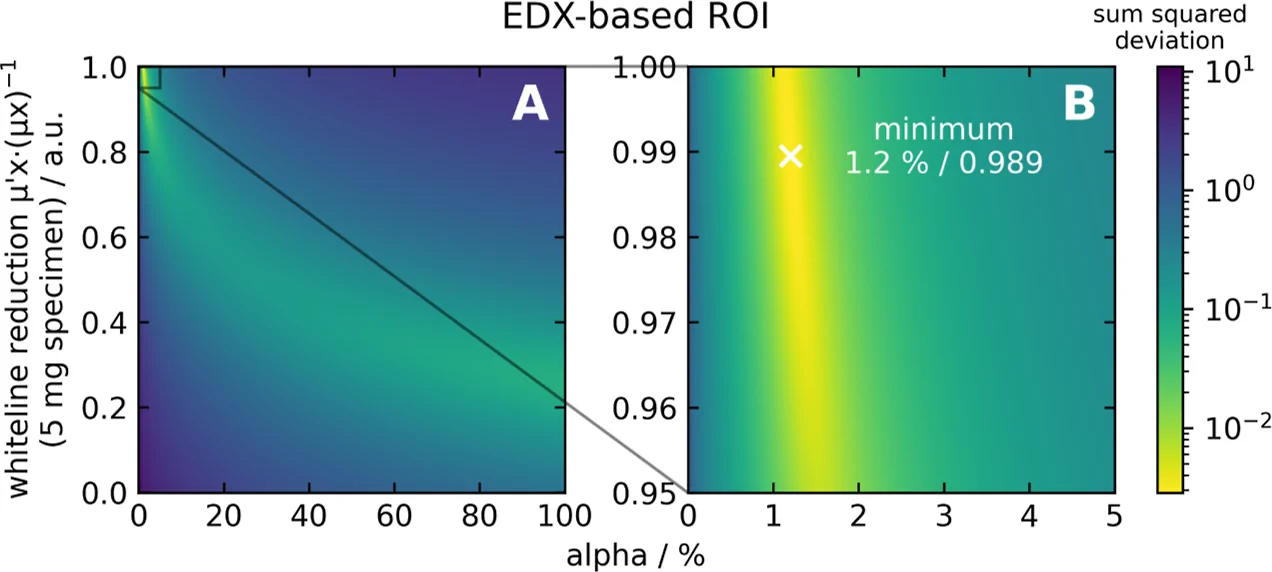
Sum squared deviation for EDX-based spectrometer with ROI evaluation. The 5 mg specimen whiteline reduction is varied along the alpha factor and the sum squared deviation is calculated from the resulting data and the theoretic values after McMaster et al. (as shown in Table 2). Panel A denotes the full evaluated range, and panel B shows the zoom around the minimum.

**Table 3:** Optimal Fit of the Whiteline Reduction to a Respective Leak Fraction Alpha^a^ ^b^

best fits for the whiteline reduction factor			
instrument setting	alpha/%	whiteline reduction at 5 mg	deviation
EDX-ROI	1.2	0.989	0.0028
EDX-Fit	0.7	0.997	0.0022
2D-detector	16.6	0.813	0.0040
EDX-all^b^	186	0.279	0.0012

a Given are the best fitting leak fraction alpha, the whiteline reduction of the 5 mg specimen, and the deviation from the alpha function as summed squared deviations.

b Data for EDX—all do not derive from a best fit but from the count ratio in the EDX spectra.

Plotting the reduction of the whiteline (absorbance) obtained from the best fit as described above over the true absorbance and in the experimental apart (eq 12) allows a comparison with calculated curves representing different leak fractions (lines in Figure 7). The whiteline reduction obtained with conventional settings of the EDX-based and 2D-detector instruments as well as for two non-conventional processing procedures of the spectra from the EDX-based instrument are shown in Figure 7 by geometric shapes. Note that the EDX-based instrument all spectrum data were fitted to 186% alpha, which was derived from the ROI and the total counts of the EDX spectrum in the whiteline region.

**Figure 7:**
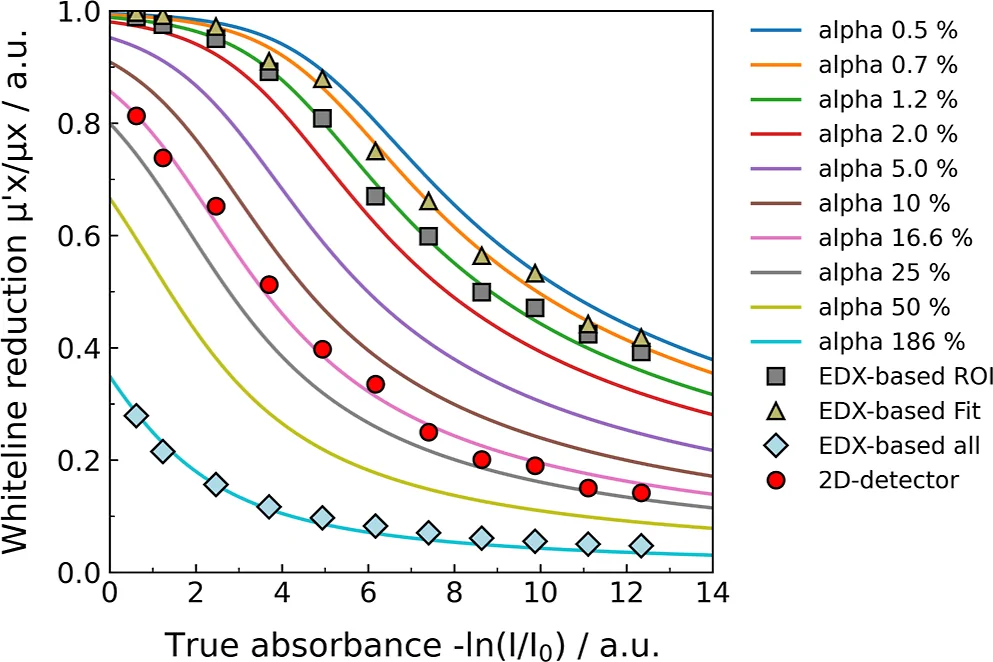
Reduction of absorbance increasing with the true absorbance modeled for different leak fractions and experimentally determined using the best fits for the 5 mg sample whiteline reduction.

### Leak Radiation Sources

The sources of radiation not absorbable by the sample can be manifold. Scatter, fluorescence, higher harmonics, and monochromator energy spectrum, i.e., wing function have been discussed as possible sources.^33,36,38^ The influence of a wing function of the energy and a leak fraction constant over the entire energy regime with increasing sample thickness was modeled, as described in the experimental section. The results show that a wing function of the energy spectrum leads to a distortion of the absorption at the edge, while a constant leak fraction results in an overall damping of the absorption. The simulation of spectra modeled for specimen with an increasing number of layers, i.e., thickness and an energy wing function is shown in Figure 8a, the asymmetric damping at the main edge with increasing thickness is well visible. A similar kind of damping has been observed from spectra obtained with a mosaic crystal at the TU Berlin in a laboratory-XAFS instrument operating a Highly Annealed Pyrolytic Graphite (HAPG) crystal.^46–48^ The spectra obtained here from the Ge(400) and Si(220) crystals do not show a comparable distortion. Hence, it can be concluded that the energy function from both crystals is not a significant source of leak radiation. In Figure 8b, the simulations of XANES spectra applying a constant leak fraction are shown. Here a constant damping with increasing number of layers is visible. This damping matches well with the damping observed in the experiments for both instruments used here.

**Figure 8:**
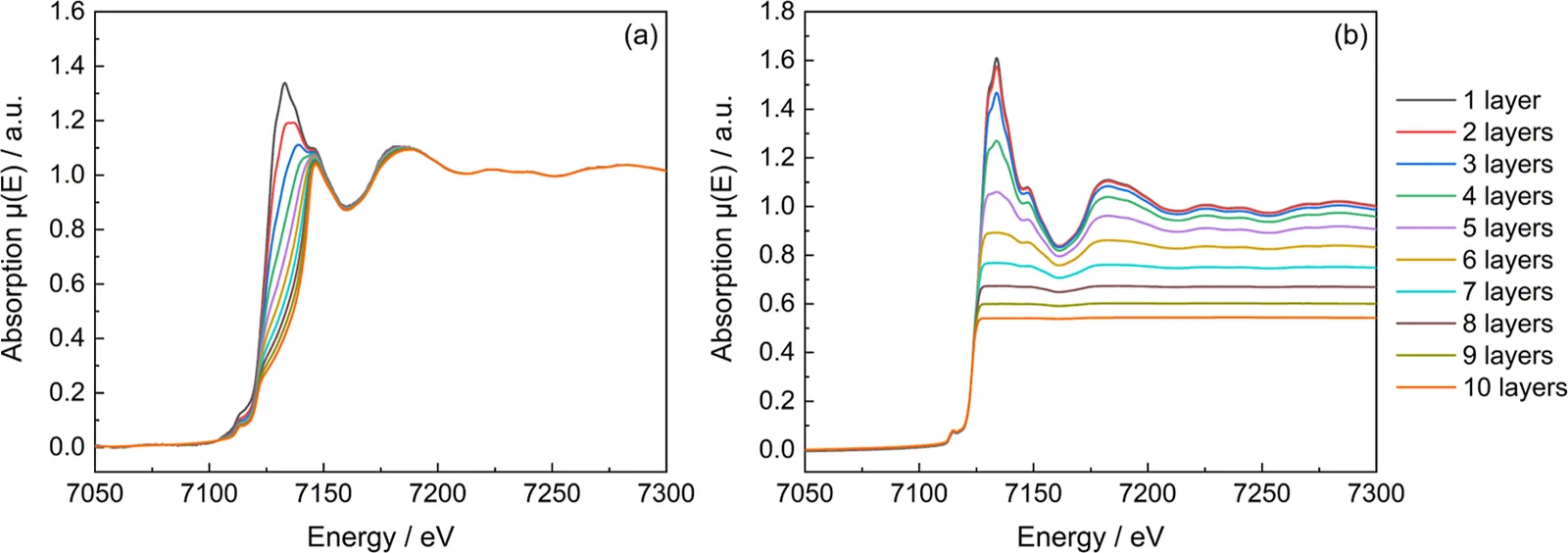
(a) Simulated Fe K-edge absorption spectra of Fe_2_O_3_ assuming an energy wing function of I_0_ (length of window tail = 50 eV) and increasing numbers of layers and (b) simulated Fe K-edge absorption spectra assuming a constant leak fraction of *I*_0_ and increasing numbers of layers.

The source of the constant leak fraction could be fluorescence, scatter, and higher harmonics. While the data from the EDX-based spectrometer use an ROI to build the spectra, excluding higher harmonics and fluorescence, the 2D-detector instrument uses a broader energy spectrum. This could explain the higher leakage fraction determined for this instrument. To obtain information on the influence of scatter and higher harmonics, the energy-dispersive spectra from the EDX-based instrument were reevaluated by (a) using the background-subtracted and fitted monochromator reflection and (b) using the entire spectra, including all higher harmonics. An EDX spectrum normalized to the exposure time is shown in Figure 9. The commonly used ROI is indicated with the blue line, the fit with the red line, and the entire spectrum within the green lines. The fitting routine was established in Python by using the summed spectra of each measurement point. A Gaussian distribution was assumed for the corresponding peak with a linear background in the first approximation within 5 and 7.5 keV.

**Figure 9:**
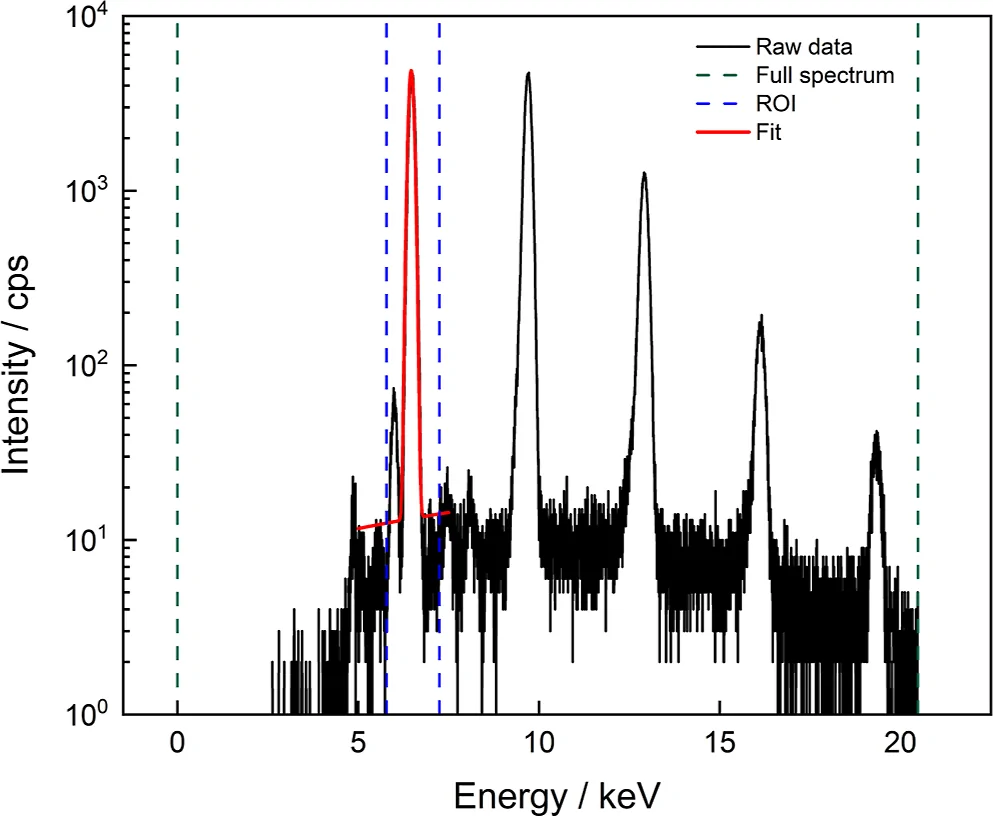
Merged EDX spectrum normalized to exposure time obtained from ten individual measurements of 1 s. For XANES acquisition, either a ROI (between blue lines), the full spectrum (between green lines), or a fit of the peak (in red) can be chosen.

The results show a lower damping when the fitted spectra are used and an increase of damping with the full spectrum including higher harmonics, scatter, and fluorescence. A distortion resulting from a monochromator wing function or Johann error cannot be observed. The spectra are shown in Figure 10. Including the data into the diagram of the whiteline reduction (Figure 7; gray triangles and blue diamonds), the fitted spectra match a leak fraction of 0.7% best, while the data processing using the entire spectrum leads to a leak fraction of 186%.

**Figure 10:**
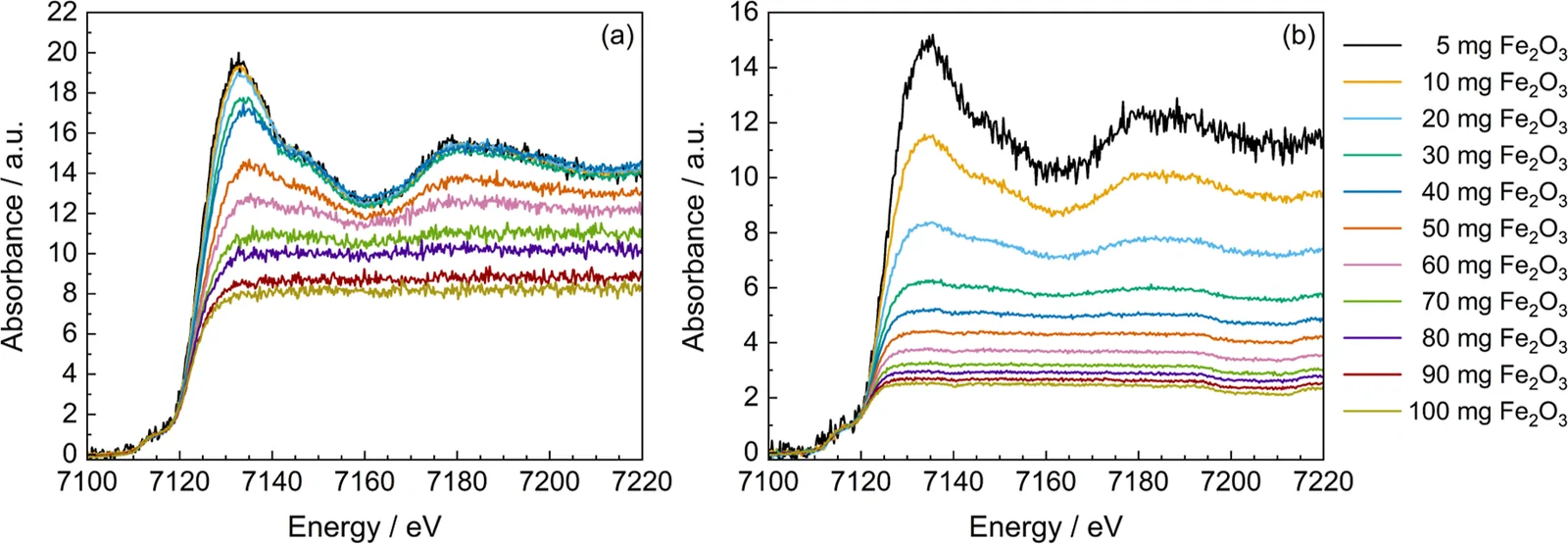
Fe K-edge absorption spectra and are normalized to the pre-edge (a) obtained with the fitted reflection and (b) obtained from the entire EDX spectra. Measurements were performed with the EDX-based instrument.

In conclusion, removing the background and fitting the reflection reduced the leak fraction by about a half percent compared to the data processing using the ROI.

The amount of leak flux has implications for the necessity to prepare thin samples. As seen in the spectra in Figures 4 and 10, low masses and thin samples will yield acceptable low errors due to damping. In the example shown here, the preparation of 10 mg Fe_2_O_3_ is optimal for the EDX-based instrument (corresponding to ca. 7 mg of Fe) as it provides the lowest damping. The 5 mg specimen is difficult to prepare homogeneously and suffers from higher statistical noise. With respect to the minimum statistical noise, the 20 mg specimen measured with the EDX-based instrument yields even lower noise but higher damping (Figure 5 and eq 12).

### Errors in Linear Combination Fits

The damping of features in the near-edge absorption spectrum can significantly affect the determination of the species composition in a multi-component sample. Linear combination fitting is a versatile tool for identifying the species composition present in a sample. The references used for the fit must match the species present in the sample well. The references and samples should be prepared reasonably thin to prevent damping. In the research on slag valorization and recycling of critical elements, the characterization of the compounds formed is fundamental. Redox-active elements such as Fe form different compounds depending on the environmental conditions and their oxidation state. For example, Fe^2+^ and Fe^3+^ frequently form spinel solid solutions, especially when Cr and Mg are present, which can be advantageous in recycling magnetite from EAF slags. Accordingly, Fe species in slags from the EAF serve as examples to explore the influence of damping on species determination.

The EAF slags are quite rich in Fe. The slag used here contains 312 ± 1 mg·g^–1^ of iron determined by ICP-OES measurements. XRF spectrometry was used to determine the bulk composition of the slag; the results can be seen in Table 4.

**Table 4:** Main Components of the EAF Slag Determined by XRF Spectrometry^a^

compound	mass concentration/wt %
MgO	4.0
Al_2_O_3_	9.1
SiO_2_	12.5
CaO	21.8
MnO	5.4
Fe_2_O_3_	42.9
Cr_2_O_3_	3.0

a They sum up to 98.8 wt.-%; the remaining 1.2 wt.-% consists of minor components such as sulfur, vanadium, copper, and zinc.

The determined mass concentrations of the main components served as basis for calculating the desired slag masses to be used in the pellets according to the Beer–Lambert’s law (eq 4). The specimens were prepared with 8, 20, 30, 41, 50, 60, 70, and 80 mg of the EAF slag, corresponding to a theoretical absorbance of 0.8, 2.0, 3.0, 4.1, 5.0, 6.0, 7.0, and 8.0. The XANES spectra are shown in Figure 11.

**Figure 11:**
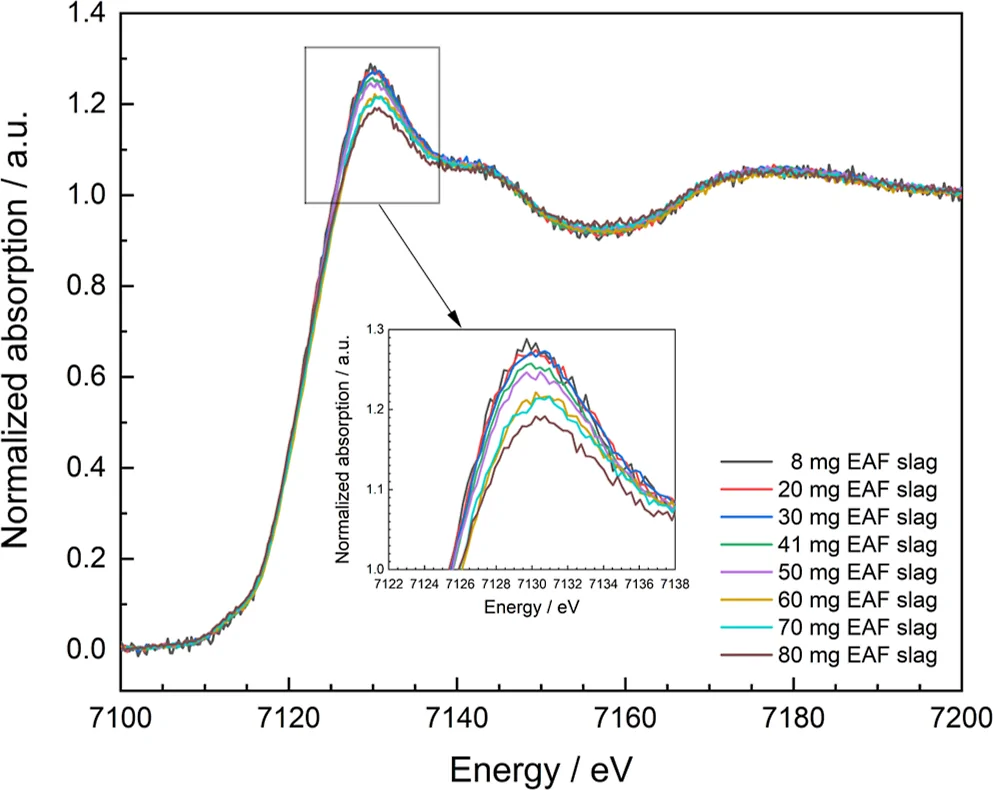
Fe K-edge XANES of EAF slags from specimens of different mass concentrations.

It is well visible that specimens with a higher mass load show higher damping in the XANES. The damping is mostly visible in the whiteline; however, the 80 mg spectra also exhibit a dampened amplitude. Through LCF with suitable reference compounds, the species and fractions in the slag sample can be determined.

Reference compounds for the LCF should be selected with care. This is especially important when investigating a complex sample composition with unknown (mineralogical) phases. It is essential that these (mineralogical) phases are determined by complementary methods beforehand so that a sensible set of reference materials can be employed in the LCF.

Here, the EAF slag was investigated regarding its mineralogical phases with Powder X-ray Diffraction (PXRD) and Electron Probe Microanalysis (EPMA) as complementary methods in order to select the reference compounds comprehensively. PXRD strongly supported the presence of gehlenite, iron-containing chromite, wüstite, calcite, and larnite (Figure S8 and Table S5). EPMA on single grains confirms the presence of gehlenite, iron-containing chromium spinel, and silicate phases (potentially with Fe) but shows additionally the occurrence of magnetite. The corresponding BSE­(Z) images can be seen in Figure 12.

**Figure 12:**
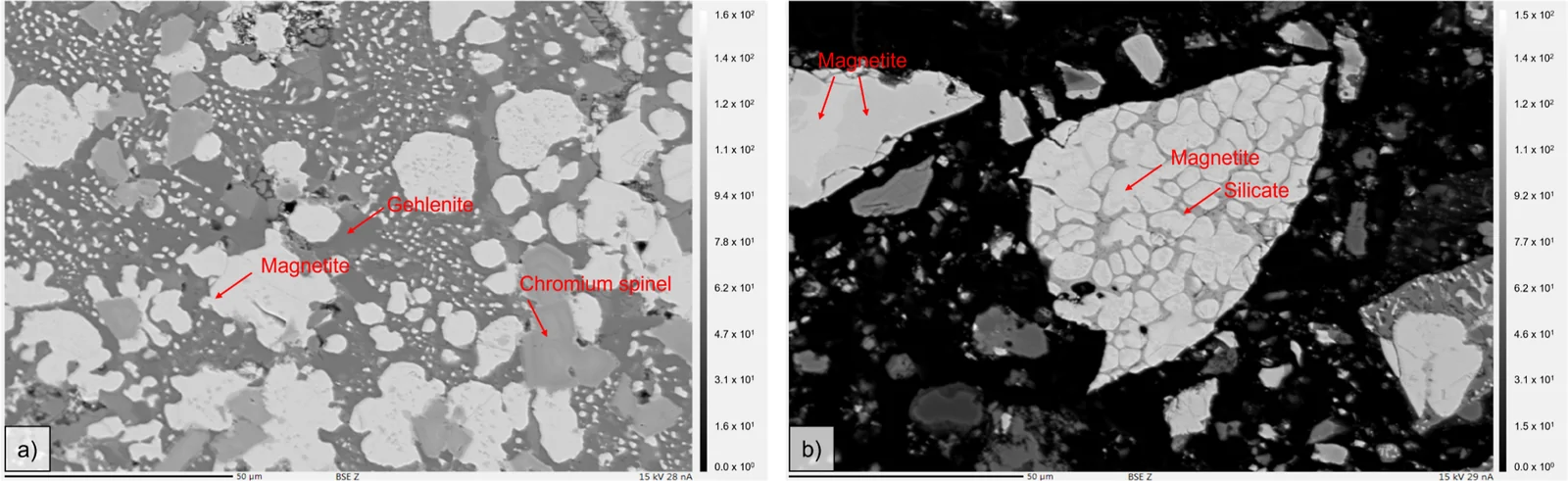
BSE­(Z) images (a) and (b) from EPMA measurements at individual spots on the as thin-section-prepared EAF slag. Identified phases are noted in red.

Hence, the references included in the LCF for a first approach were chosen to be magnetite (Fe_3_O_4_), olivine with a major compound of Fe_2_[SiO_4_], and iron chromite (FeCr_2_O_4_). Wüstite was not available as the reference material due to its instability at room temperature.^49,50^ The LCF was applied in a region of −20 eV to +80 eV around the edge position at approx. 7122 eV in the slags spectrum and was not forced to sum the fitted fractions up to 100%. The fractions of the references were used to calculate the percentages of the corresponding iron species. Figure 13 depicts the recorded spectra together with the fits and the corresponding residuals for the 8 mg specimen as well as one 20 mg and 80 mg specimen, respectively. The spectra of the repetitively prepared and measured samples are shown in the Supporting Information (Figures S9 and S10). The results of the LCFs are given in Table 5.

**Figure 13:**
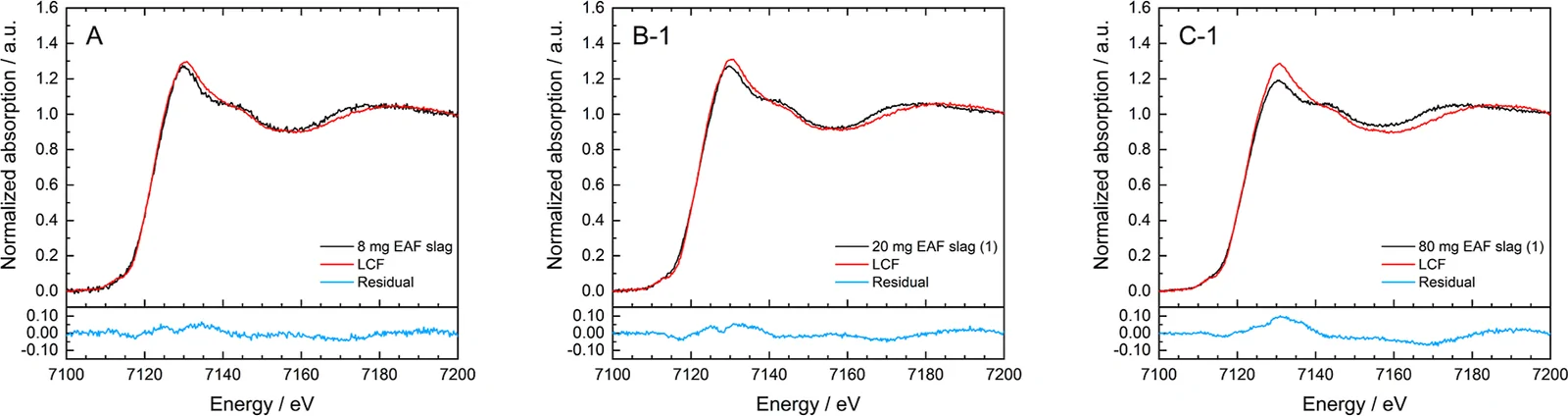
Recorded spectra of (A) 8 mg, (B-1) 20 mg, and (C-1) 80 mg specimens with fit and residuals from the LCF. This fit included magnetite, olivine, and iron chromite as reference materials.

**Table 5:** Results of the Linear Combination Fit of the EAF Slag of Different Sample Absorbance (The Mass of Slag Prepared in the Pellets is Given), Errors are Confidence Intervals (*N* = 3)^a^

iron species	8 mg	20 mg	80 mg
Fe(II) in %	65	67 ± 11	69 ± 9
Fe(III) in %	32	31 ± 10	29 ± 9
sum in %	97	98	97

a Employed reference materials were magnetite, olivine and iron chromite.

The depicted LCFs show that the pre-edge and edge regions of the XANES spectra are well fitted for all specimens. Deviations, clearly shown by the residuals, are observed for the entire spectrum. This can be explained by the complex mineralogical composition of the slag, which is challenging to be perfectly represented by the employed reference materials. In addition, higher residuals are present in the samples with higher masses, mostly at the whiteline, here the damping of the absorption has a negative impact on the fit quality. However, the LCF results (Table 5) show that the species fractions for all three chosen slag masses in the pellets are quite similar. This indicates that the observed damping for the 80 mg specimens does not significantly influence the obtained LCF results—at least if the given set of reference materials is employed.

On the basis of the observed damping for the 80 mg specimens, it can be expected that the fit results change if a reference material that has a less pronounced whiteline is provided, since it would be incorporated preferably by the fitting algorithm. In general, K-edge XANES spectra of elemental 3d metals do not show intense whitelines due to the limited final states allowed for the 1s to 4p electron transition.

A combinatorial analysis with a set of reference materials extended with hematite (Fe_2_O_3_) and metallic iron (Fe^0^) was performed (a) to prove the assumption of a preferable incorporation of metallic iron due to the damping and (b) to check which combination leads to the mathematically best fits for each specimen. These best fits were indicated by the R factor under consideration of the lowest single error relative to the respective compound. Since it is not unusual for EAF slags to contain hematite and metallic iron due to their production process,^51,52^ these components represent sensible additional reference materials to be used in the LCF for testing purposes, even though they were not explicitly found with PXRD and EPMA measurements. In Table 6, the results from the LCF with the extended set of reference materials are listed. The *R*-factors of the LCF with this set are approx. 0.4× lower in average than the *R*-factors of the previous fits without hematite and metallic iron. Figure 14 shows the spectra, the fits, and the residuals for the 8 mg specimen as well as for the 20 mg and 80 mg specimens, respectively. The figures for the repetitively prepared and measured samples are shown in the Supporting Information (Figures S11 and S12). Figure S13 shows the XANES spectra of all considered iron reference materials.

**Table 6:** Results from the Best Linear Combination Fits of the EAF Slag of Different Sample Absorbance (The Mass of Slag Prepared in the Pellets is Given), Errors are Confidence Intervals (*N* = 3)^a^

iron species	8 mg	20 mg	80 mg
Fe(0) in %	14	15 ± 3	26 ± 9
Fe(II) in %	45	46 ± 7	31 ± 3
Fe(III) in %	38	37 ± 9	40 ± 5
sum in %	97	98	98

a Employed reference materials were magnetite, olivine, iron chromite, hematite, and metallic iron.

**Figure 14:**
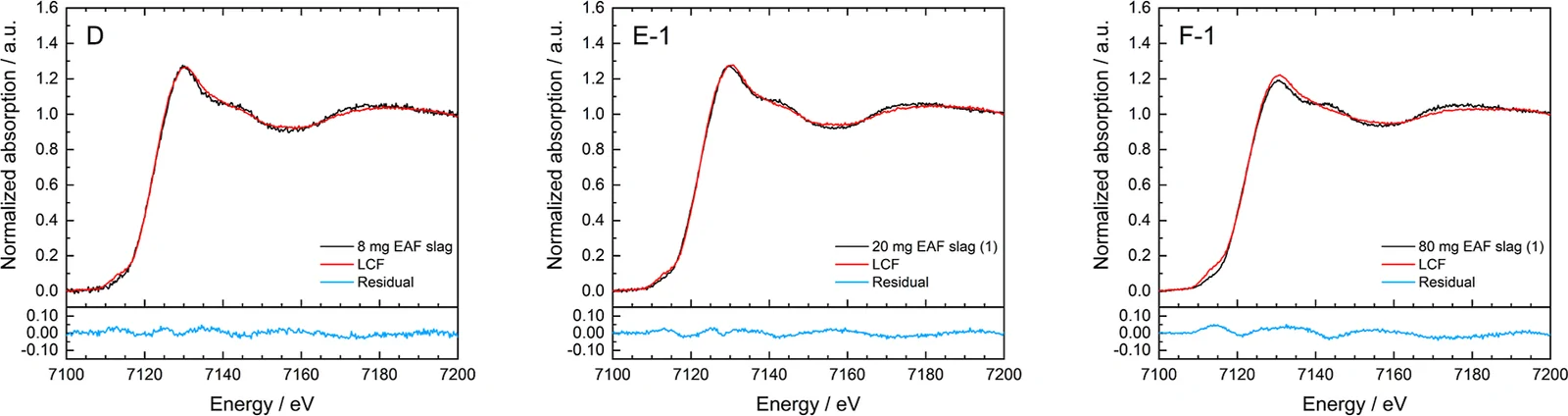
Recorded spectra of (D) 8 mg, (E-1) 20 mg, and (F-1) 80 mg specimen, with fit and residuals from the mathematically best LCF. This fit included magnetite, olivine, iron chromite, hematite, and metallic iron as reference materials.

In the corresponding LCFs of the specimens, metallic iron is incorporated while the iron chromite fraction is almost totally suppressed. The resulting species fraction of the 8 mg and 20 mg samples are quite similar. Larger differences are evident when comparing both to the 80 mg sample. The fraction of Fe­(II) is reduced by ca. 15%, and the fraction of metallic iron increases by about 12%. This over-interpretation of the metallic iron in the thick specimens most likely originates from the damping due to leak flux and leads to a fit that is less deviating from the recorded spectra in the post-edge region. However, the fits have a higher inaccuracy in the pre-edge region. When comparing the first-fit approach (Table 5) with the—from a mathematical point of view—best fit (Table 6), it can be seen that the fraction of Fe­(II) species is significantly decreased in general and even more so for the 80 mg sample. The fraction of Fe­(III) is slightly increased, but this trend is not significant due to the large confidence intervals. If the plausibility of the fits is concerned, it can be stated that the fit incorporating magnetite, olivine, and iron chromite is more sensible and experimentally more verified. A resulting amount of metallic iron greater than 10% would not be unusual according to the literature^52^ but is certainly questionable when the iron chromite fraction is almost completely suppressed at the same time. Besides, the results from the PXRD and EPMA measurements do not show any evidence of metallic iron in the sample. A content of approx. 15% metallic iron would be visible in the PXRD diffractogram due to the specific peaks at 2θ values of approx. 45°, 65°, and 82°.^53,54^ Furthermore, particles of metallic iron would have been identifiable in the BSE­(Z) images from the EPMA measurements as bright spots with sharp contrast to the surroundings.^55^ These results emphasize the importance of a comprehensive selection of reference materials included in the LCF. Furthermore, they show that the mass concentration has an impact on the speciation analysis if damping effects occur and if a reference component with low amplitudes in the post-edge region of its XANES spectrum is involved, simultaneously. Especially, interpretation should not only be based on the R-factors of the LCF, since these values only reflect a mathematical goodness of the fit and do not consider the plausibility of the employed references.

## Conclusion

With this study, valuable insights were gained about the influence of the sample thickness on the damping of Fe K-edge spectra and the influence of the sample absorbance on the errors in the linear combination fits. The investigation of Fe K-edge spectra obtained from two laboratory-based X-ray absorption spectrometers showed that a constant leak fraction fits the data very well. The energy-dispersive spectrum used in one of the spectrometers showed, besides the first-order reflection, the additional presence of higher-order reflections and fluorescence lines (Figure 9). Including all of those in the fit increases the damping of the spectra, as expected from the models. Subtracting the background from the first-order reflection, which is usually not done in the routine, reduces the damping and improves the quality of the spectra so that there is more leeway for sample preparation regarding the absorbance. An EAF slag served as a complex model substance for investigating the impact of the damping on the iron speciation through XANES. Characterization of the slag regarding its iron content and its mineralogical phases was done by ICP-OES with previous wet-chemical digestion, XRF, PXRD, and EPMA. With an appropriate set of reference material (consisting of magnetite, olivine, and iron chromite) verified by the methods, the determination of iron species in the slag was not significantly influenced by the damping among the specimens. Careful interpretation is necessary if an Fe^0^ component is added to the fit. Due to the lower amplitudes in the post-edge region, it is possible that this fraction is overestimated, especially in the dampened spectra.

## Supplementary Material



## Notes

The Supporting Information are available free of charge. The Supporting Information is available free of charge at https://pubs.acs.org/doi/10.1021/acs.analchem.6c02072.

Wet-chemical digestion, ICP-OES measurements, XANES measurements, pellet preparation, assumptions for the 5 mg specimen, iron speciation, and the recorded diffractogram of the PXRD analysis alongside details about its evaluation (PDF)

## §

Deutsches Elektronen-Synchrotron DESY, Notkestrasse 85, 22607 Hamburg, Germany
