# Supplementary material for: Errors in the Laboratory X‑ray Absorption Near Edge Structure with Respect to Species Analysis in Slags Considering Leak Radiation
Source: Anal Chem. 2026 Sep 30;98(40):29174–87. doi: 10.1021/acs.analchem.6c02072 (PMC13670889; doi:10.1021/acs.analchem.6c02072)
Supplement: Supplementary file 1 [file ac6c02072_si_001.pdf]

Errors in laboratory X-ray absorption near edge structure with respect to species analysis in slags considering leak radiation

Jessica Hiller,[†] Joanna Kolny-Olesiak,[†] Sven Hampel,[†,¶] Thomas Schirmer,[‡] and Ursula E. A. Fittschen[*,†]

†*Institute of Inorganic and Analytical Chemistry, Clausthal University of Technology, 38678 Clausthal-Zellerfeld, Germany*

‡*Institute of Geotechnology and Mineral Resources, Clausthal University of Technology, 38678 Clausthal-Zellerfeld, Germany*

¶*current address: Deutsches Elektronen-Synchrotron DESY, Notkestrasse 85, 22607 Hamburg, Germany*

E-mail: ursula.fittschen@tu-clausthal.de

Phone: +49 (0)5323722205

Abstract

This supplementary material includes detailed experimental information about the wet-chemical digestion, the ICP-OES measurements, XANES measurements as well as supporting figures for pellet preparation, assumptions for the 5 mg specimen, the iron speciation and the recorded diffractogram of the PXRD analysis alongside details about its evaluation. The supplementary material is licensed under a Creative Commons Attribution 4.0 International License (CC BY 4.0).

Contents

Section **Page**

Introduction **S3**

XANES measurements of Mn containing samples S3

Experimental **S4**

Element determination in the slag material S4

Microwave-assisted digestion S4

ICP-OES measurements S5

XANES measurements S6

Fe containing samples S6

Results and Discussion **S8**

Evaluation of leak flux (alpha factor) S8

Evaluation of the true absorbance of a data set S9

Sensitivity of assumptions regarding the whiteline reduction and the true absorbance of the 5 mg specimen S9

Errors in linear combination fits S12

PXR diffractogram and its evaluation S12

LCFs and XANES spectra of the reference materials S13

References **S15**

Introduction

XANES measurements of Mn containing samples

The MnO_2 specimen with data shown in Figure 1 was prepared from manganese(IV) oxide (90 %, for synthesis) and cellulose (both Merck KGaA, Darmstadt, Germany) as described by Hampel *et al.*[1] The pellet was prepared as a pellet with 10 mg MnO_2 and 80 mg cellulose and encapsulated in self-adhesive poly(imide) foil. Figure 1 shows laboratory XANES data in accordance to previous results from Hampel *et al.*[1] and the measurement parameters are given in Table S1. Five measurements for I as well as I_0 were merged. I_0 measurements were performed on pure cellulose pressed to an encapsulated pellet.

Table S1: Scan parameters for laboratory Mn K-edge XANES.[1]

	Energy region / eV	Step size / eV	Acquisition time per step / s
Zone 1	6482 - 6739	0.25	2
Zone 2	6739 - 6899	1	1

The manganese(IV) oxide pellet was also probed at the BAM*line* endstation at BESSY II (Berlin, Germany).[2] A superconducting 7 T wavelength shifter served as insertion device. The beam was monochromatized with a Si(111) double crystal monochromator (energy resolution $\Delta E/E = 2{\cdot}10^{-4}$) and subsequently restricted to a beam size of 4 mm × 2 mm given by slits. Measurements were performed in transmission mode with separate ionization chambers for I and I_0.

Table S2: Scan parameters for synchrotron Mn K-edge XANES.

	Energy region / eV	Step size / eV	Acquisition time per step / s
Zone 1	6339 - 6509	10	1
Zone 2	6520 - 6559	0.5	1
Zone 3	6559 - 6739	1	1
	Energy region / k-space	Step size / k-space	Acquisition time per step / s
Zone 4	7.26 - 16	0.04	1

The aquisition settings are shown in Table S2. For both datasets an energy calibration with a Mn foil (ExafsMaterials) was performed and data was normalized with a Python script similar to ATHENA.[3] The calibration routine for the laboratory XANES is described by Wittkowski *et al.*[4]

Experimental

Element determination in the slag material

In the following, the performed two-step microwave-assisted digestion and the subsequent analysis of the digestion solutions is described in detail.

Microwave-assisted digestion

200 mg of the pre-ground slag (d90 < 100 µm) were weighed into a PTFE digestion vessel containing a magnetic stirring bar, which was placed beforehand. Subsequently, 8 mL of 65 % (m/v) nitric acid (p.a.; Carl Roth GmbH + Co. KG, Karlsruhe, Germany), 5 mL of 85 % (m/v) ortho-phosphoric acid (p.a.; Merck KGaA, Darmstadt, Germany) and 1 mL of 40 % (m/v) hydrofluoric acid (p.a.; Merck KGaA, Darmstadt, Germany) were added in this order. The sealed digestion vessel was placed into the lab microwave system (Discover SP-D 80, CEM corporation, USA) and the digestion program listed in Table S3 was started.

Table S3: Operating parameters of the digestion programs.

Digestion step	Program part	Temperature / °C	Ramping time / min	Holding time / min
Acid digestion	I	100	2	5
	II	200	5	—
	III	230	8	30
Complexation	I	100	10	5

After completion, 6.5 mL of a saturated aqueous solution of orthoboric acid (p.a.; Merck KGaA, Darmstadt, Germany) was added and the complexation program was carried out (see Table S3). In both digestion steps, a medium stirring intensity, a maximum pressure of 27 bar and a maximum power of 300 W were allowed. The resulted digestion solution was light-green in color and did not contain any visible residues. Finally, the solution was transferred into a 100-mL-volumetric flask made of polypropylene and the volume was filled up to the calibration mark with ultra-pure water.

ICP-OES measurements

100 µL of the obtained digestion solutions were extracted and further acidified with 72 µL of nitric acid, 45 µL of ortho-phosphoric acid and 59 µL of a saturated aqueous solution of orthoboric acid. Subsequently, the mixture was diluted with ultra-pure water to a volume of 20 mL (dilution factor = 200). Appropriate amounts of the acids employed in the digestions (except hydrofluoric acid) were added to adjust the standards to the acid concentrations in the diluted sample solutions. The calibration standards were prepared from certified single-element standard solutions (1 $g \cdot L^{-1}$; Carl Roth GmbH + Co. KG, Karlsruhe, Germany) and contained the following final iron concentrations: 0, 0.05, 0.1, 0.25, 0.5, 1, 2, 5 , 10 $mg \cdot L^{-1}$. Each measurement was automatically done in triplicate leading to a total of nine values for the slag samples and the blank samples, respectively. The measurement values were evaluated including a comparison with the limit of quantification (LOQ) based on the statistical properties of the calibration lines. Finally, the iron contents were calculated considering the

weight portion employed in the respective digestion and a statistical evaluation (outliers test according to Grubbs, calculation of the confidence interval) was performed.

XANES measurements

Fe containing samples

A photo of a subset of the pellet series containing different masses of Fe_2O_3 was taken to illustrate the appearance of the pellets and is shown in the following Figure S1.

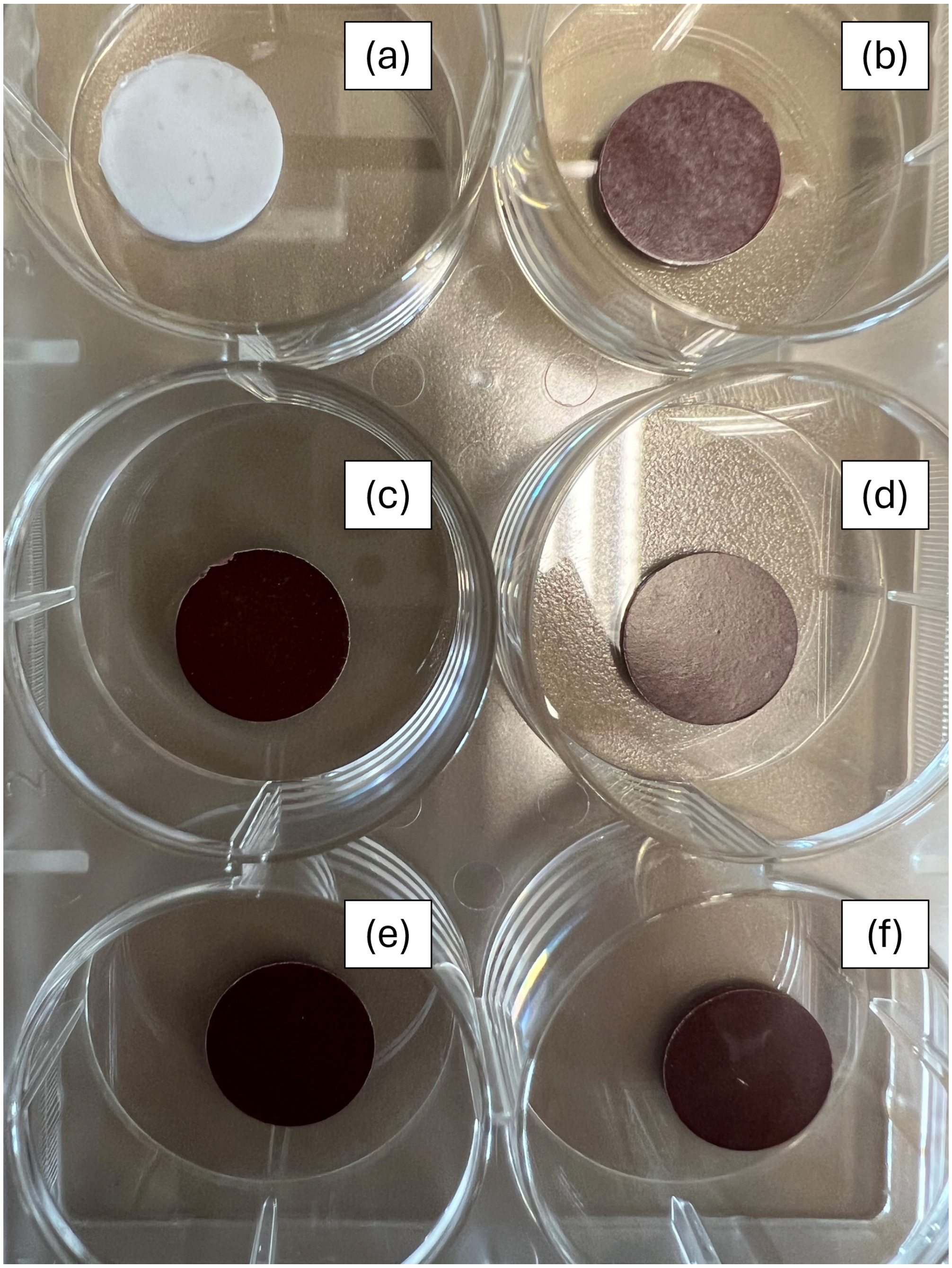

Figure S1: Photo illustrating a subset of the Fe_2O_3 pellets. Each pellet contains *approx.* 80 mg of the binder PE and an additional amount of Fe_2O_3 powder: (a) Blank pellet (0 mg Fe_2O_3), (b) 5 mg Fe_2O_3, (c) 10 mg Fe_2O_3, (d) 20 mg Fe_2O_3, (e) 30 mg Fe_2O_3, (f) 40 mg Fe_2O_3.

The reference and slag pellets were measured with the scan parameters listed in Table S4.

Table S4: Scan parameters for the quantitative investigation of the slag regarding its contained iron species.

	Energy region / eV	Step size / eV	Acquisition time per step / s
Zone 1	6930 - 7030	2.0	1
Zone 2	7030 - 7200	0.25	4
Zone 3	7200 - 7400	5.0	1

Results and Discussion

Evaluation of leak flux (alpha factor)

A series of pellets comprising different masses of Fe_2O_3 was measured with both an EDX-based and a 2D-detector spectrometer. The recorded, non-normalized XANES spectra are depicted in the following Figure S2.

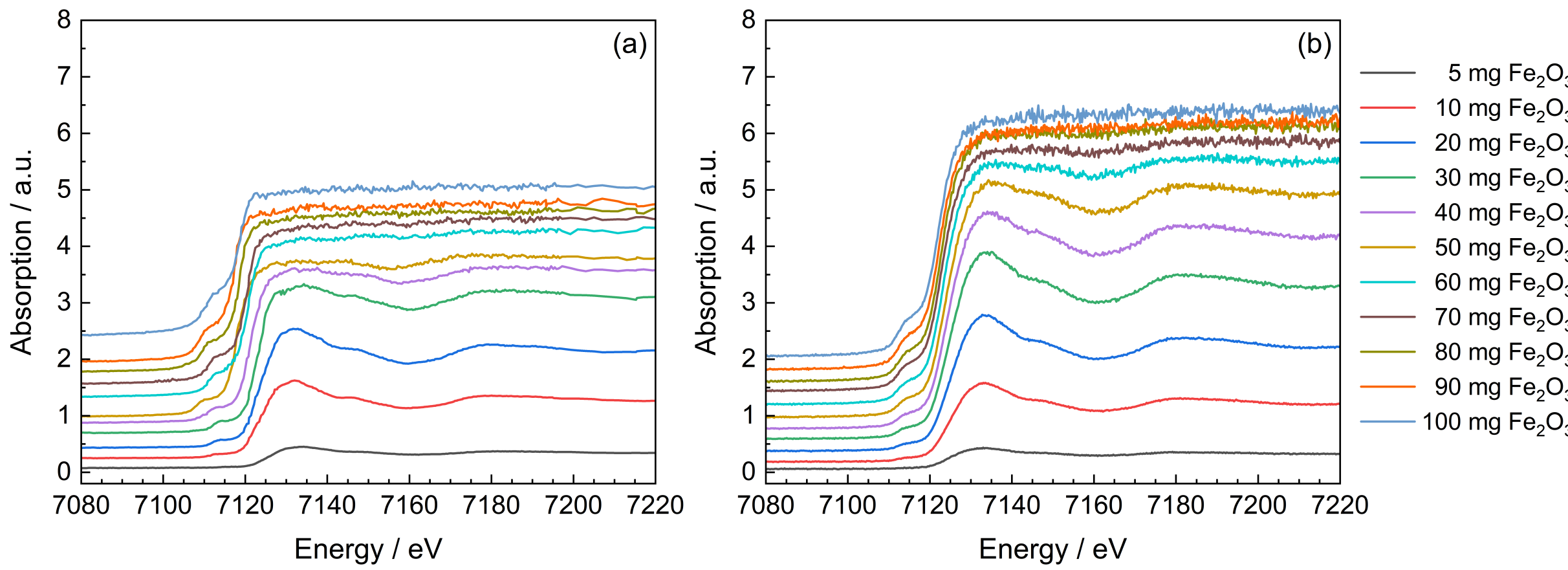

Figure S2: Non-normalized XANES spectra of pellets with different mass concentrations of Fe_2O_3 recorded with a) a 2D-detector spectrometer and b) with an EDX-based spectrometer.

Evaluation of the true absorbance of a data set

Sensitivity of assumptions regarding the whiteline reduction and the true absorbance of the 5 mg specimen

The simulated errors for different 5 mg whiteline reductions as well as the optimal alpha factor and the lowest error are shown in Figure S3 and S4 for the EDX-based instrument with fitted data and for the 2D-instrument. The simulated errors for the EDX-all spectra processing is also shown, however here no minimum was obtained (Figure S5).

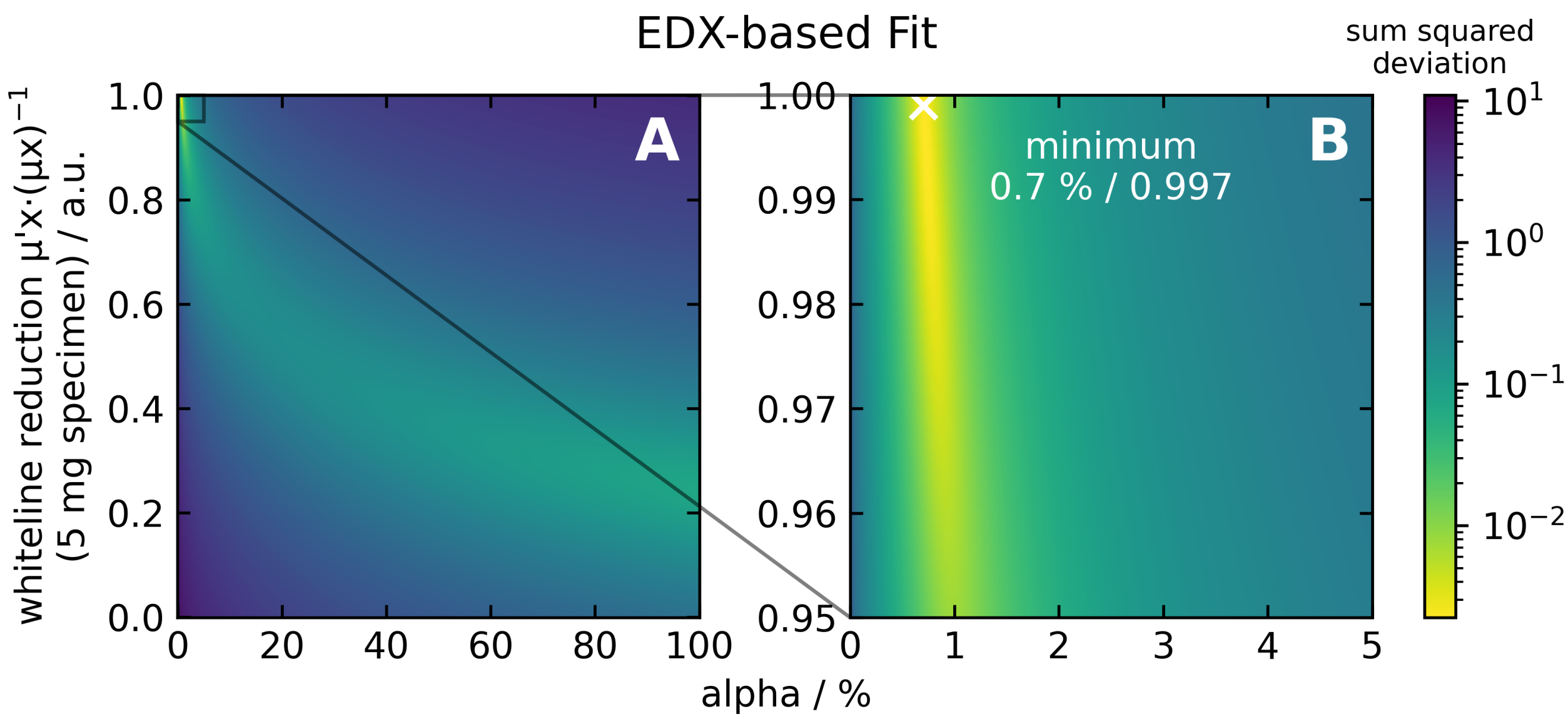

Figure S3: Sum squared deviation for EDX-based spectrometer with fit evaluation. The 5 mg specimen whiteline reduction is varied along the alpha factor and the sum squared deviation is calculated from the resulting data and the theoretic values after McMaster *et al.* (as shown in Table 2). Panel A denotes the full evaluated range and panel B shows the zoom around the minimum.

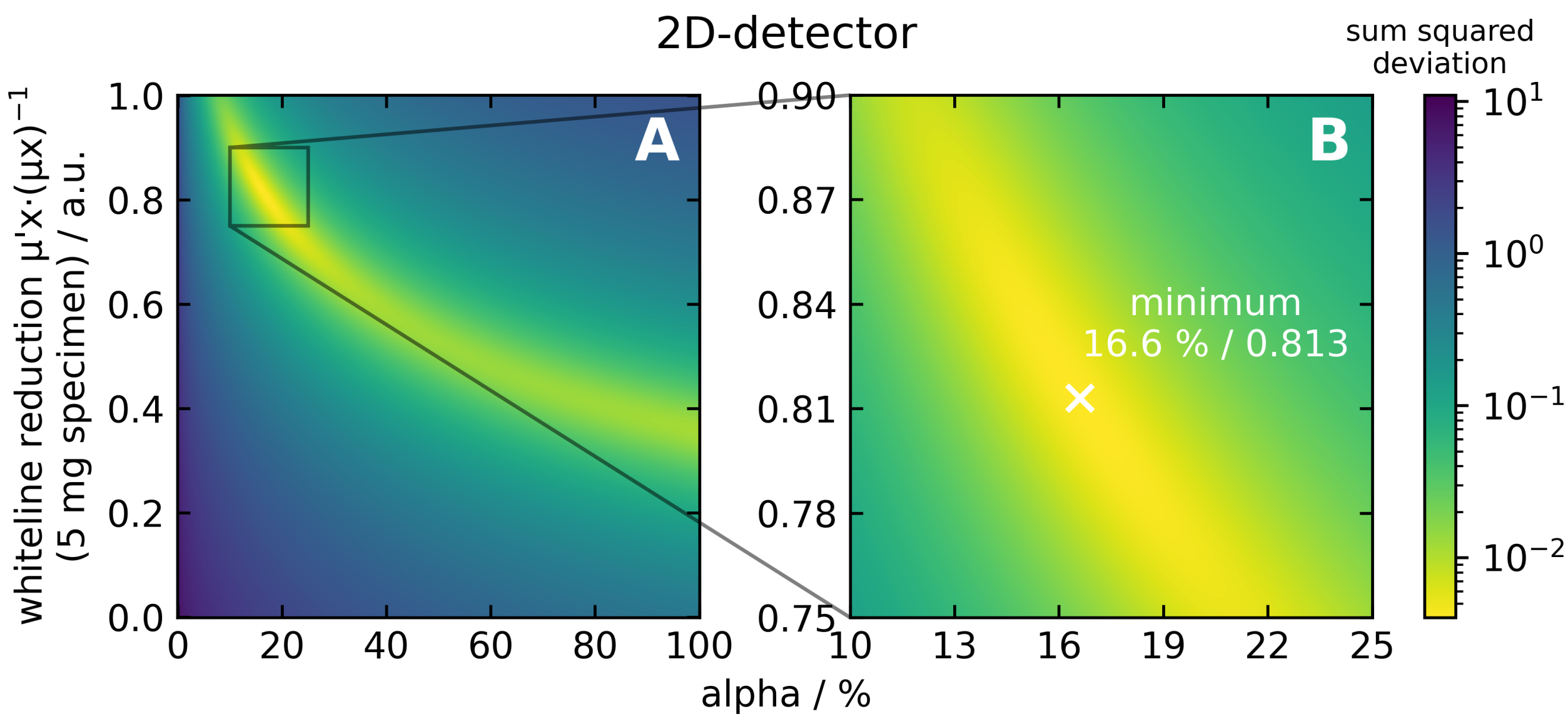

Figure S4: Sum squared deviation for 2D-detector spectrometer. The 5 mg specimen whiteline reduction is varied along the alpha factor and the sum squared deviation is calculated from the resulting data and the theoretic values after McMaster *et al.* (as shown in Table 2). Panel A denotes the full evaluated range and panel B shows the zoom around the minimum.

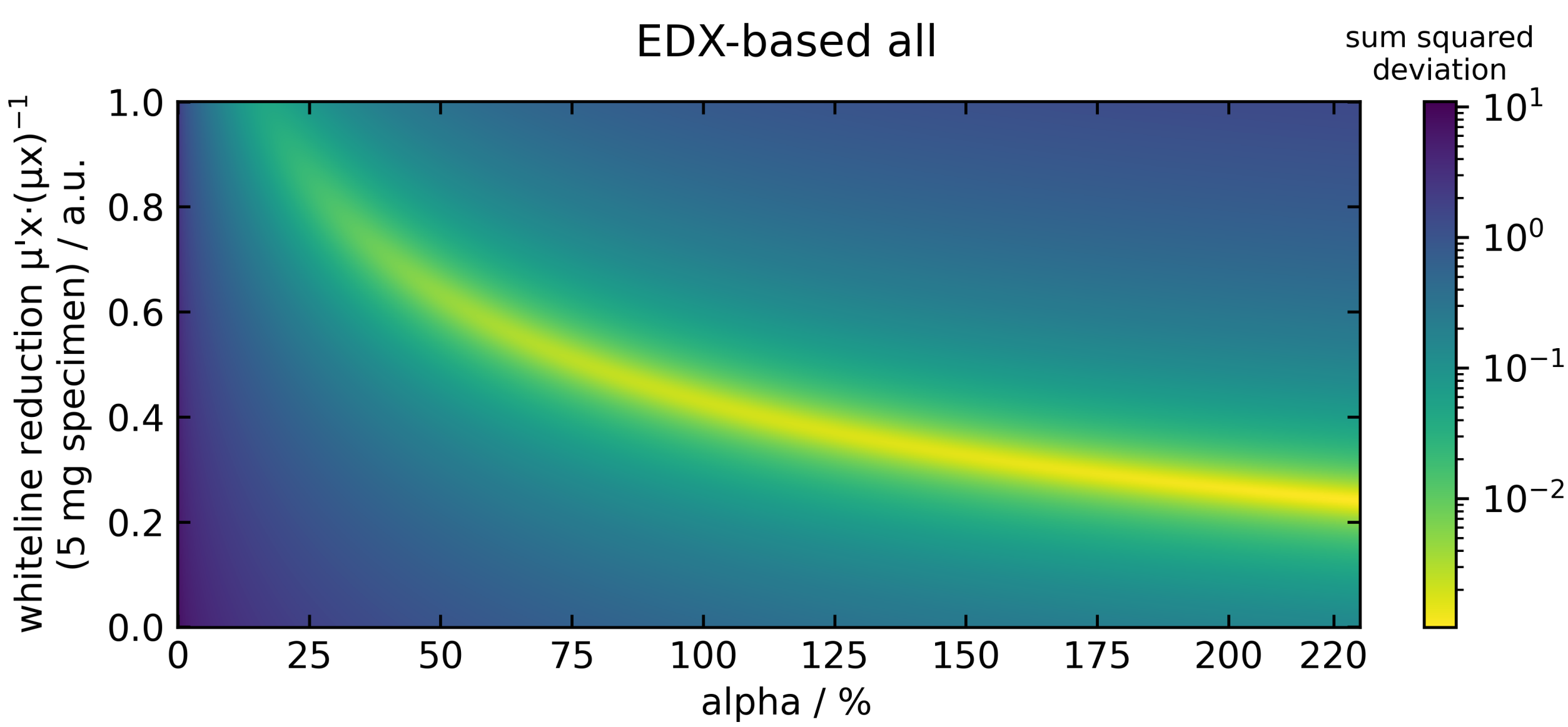

Figure S5: Sum squared deviation for EDX-based spectrometer with full-spectrum evaluation. The 5 mg specimen whiteline reduction is varied along the alpha factor and the sum squared deviation is calculated from the resulting data and the theoretic values after McMaster *et al.* (as shown in Table 2).

The following figures show the modeled whiteline reduction for different alphas and the

experimental ROI data from the EDX-based instrument normalizing the whiteline reductions to different factors achieved by the 5 mg specimen (100 %, 98.9 %, 95 %, 90 %, 80 %, 50 %). In Figure S6, a true absorption of 0.61 based on the data from McMaster *et al.*[5] was considered and in Figure S7 a true absorbance of 0.3, which is the one that was obtained by measurement for the 5 mg specimen, was used.

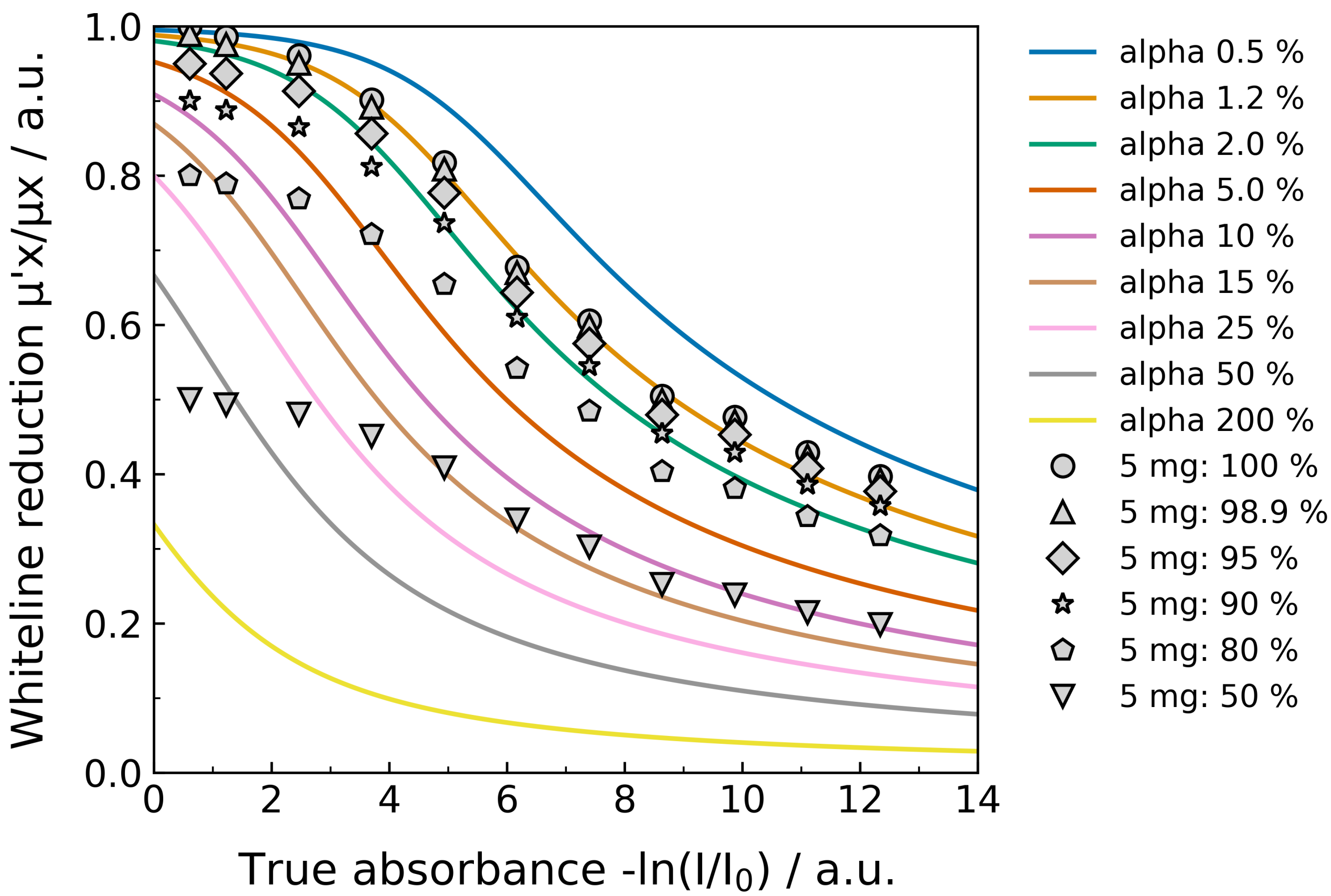

Figure S6: Reduction of absorbance increasing with the true absorbance modeled for different leak fractions considering a true absorption of 0.61.

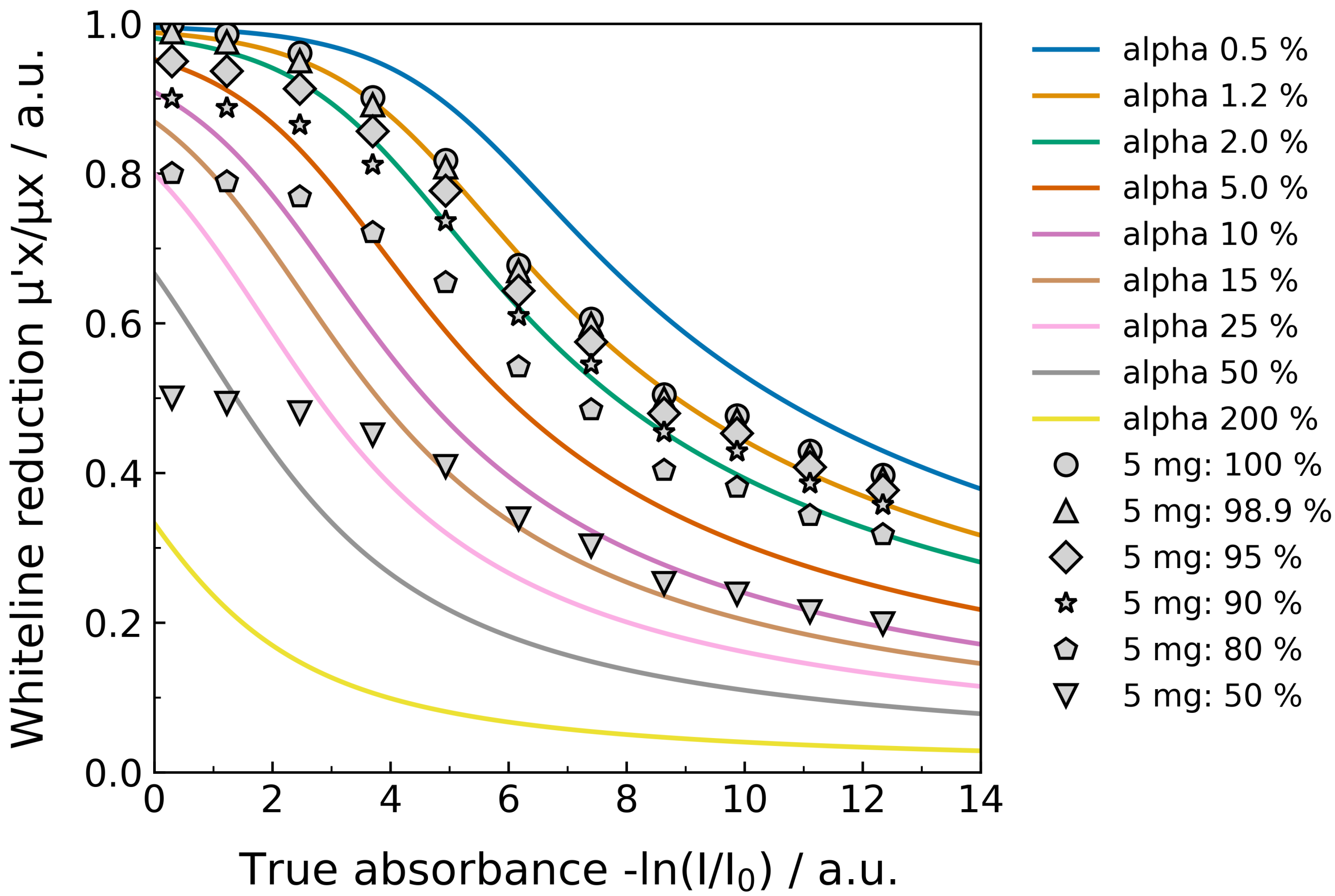

Figure S7: Modeled and experimentally derived whiteline reduction curves with a true absorbance of 0.3 as measured in the experiments.

Errors in linear combination fits

PXR diffractogram and its evaluation

The Figure S8 shows the recorded and within the software X'Pert HighScore (Malvern Panalytical, Worcestershire, United Kingdom) evaluated diffractogram of the EAF slag.

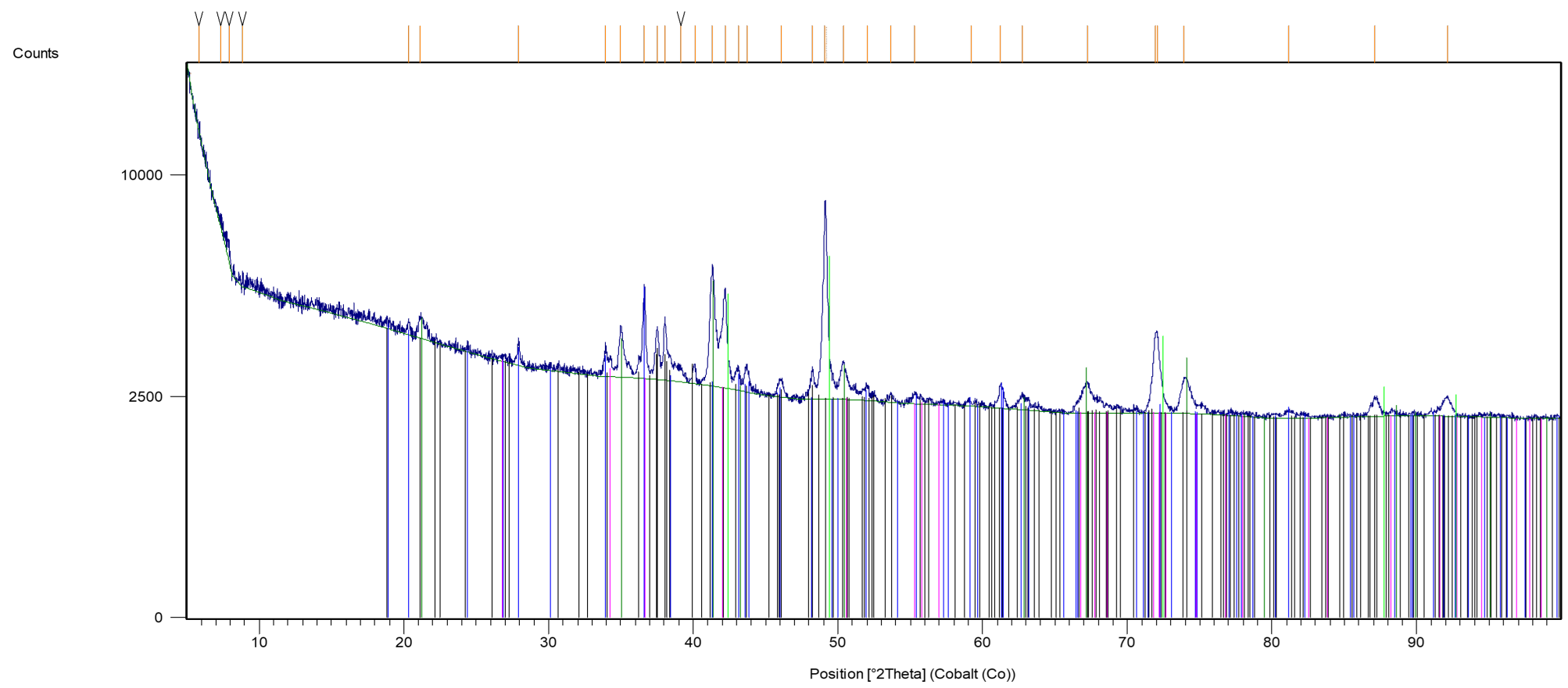

Figure S8: Recorded and evaluated PXR diffractogram of the EAF slag. Peak positions of the references are displayed in the colors listed in Table S-5.

Mineralogical phases were identified by analyzing the conformity with references from the database pdf-2 ICCD XRD.[6] The matching references are listed in Table S5.

Table S5: Identified mineralogical phases.

Reference code	Mineral name	Chemical formula	Score	Display color
01-074-1607	Gehlenite	$Ca(Al(AlSi)O_7$	40	Blue
01-072-2493	Chromite	$(Mg_{0.43}Fe_{0.58})_8(Cr_{1.19}Al_{0.77}Ti_{0.03})_8O_{32}$	35	Olive
01-074-1880	Wüstite	$Fe_{0.9536}O$	23	Lime
01-083-1762	Calcite	$Ca(CO_3)$	13	Fuchsia
01-083-0460	Larnite	$Ca_2(SiO_4)$	39	Black

LCFs and XANES spectra of the reference materials

Slag pellets contained masses of 8 mg, 20 mg and 80 mg electric arc furnace (EAF) slag, respectively. For the formulation with 20 mg and 80 mg EAF slag, pellets were prepared and measured in triplicate. The Figures S9 - S12 show the recorded spectra of the 20 mg and 80 mg specimens with the corresponding linear combinations fits and the residuals for the two fit approaches, which differed in the included set of reference materials.

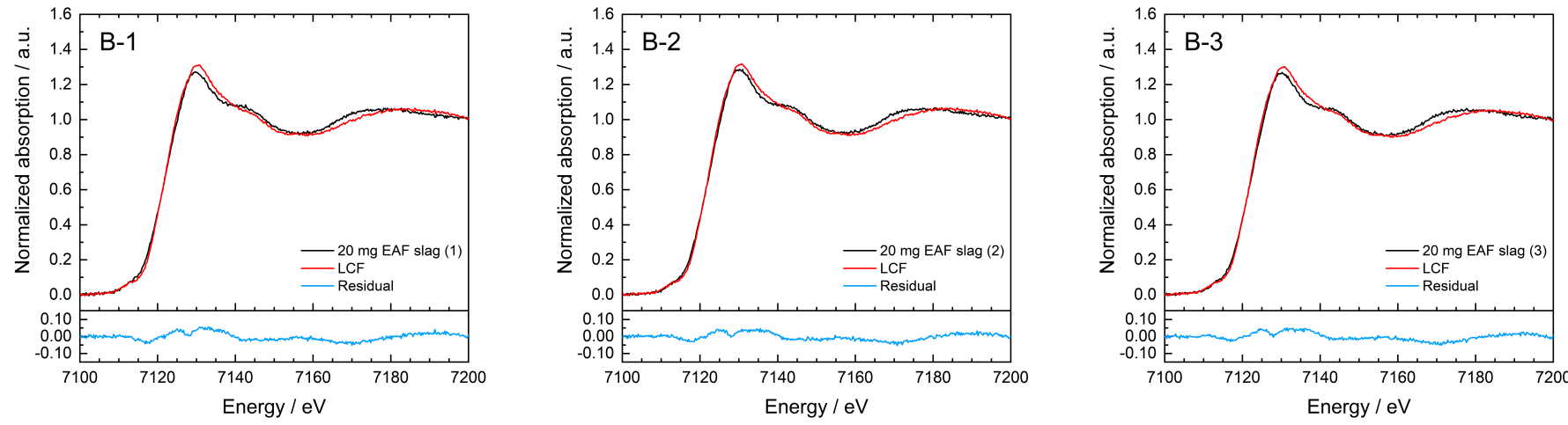

Figure S9: Recorded spectra of the three 20 mg specimens with fit and residuals from the LCFs. These fits included magnetite, olivine and iron chromite as reference materials.

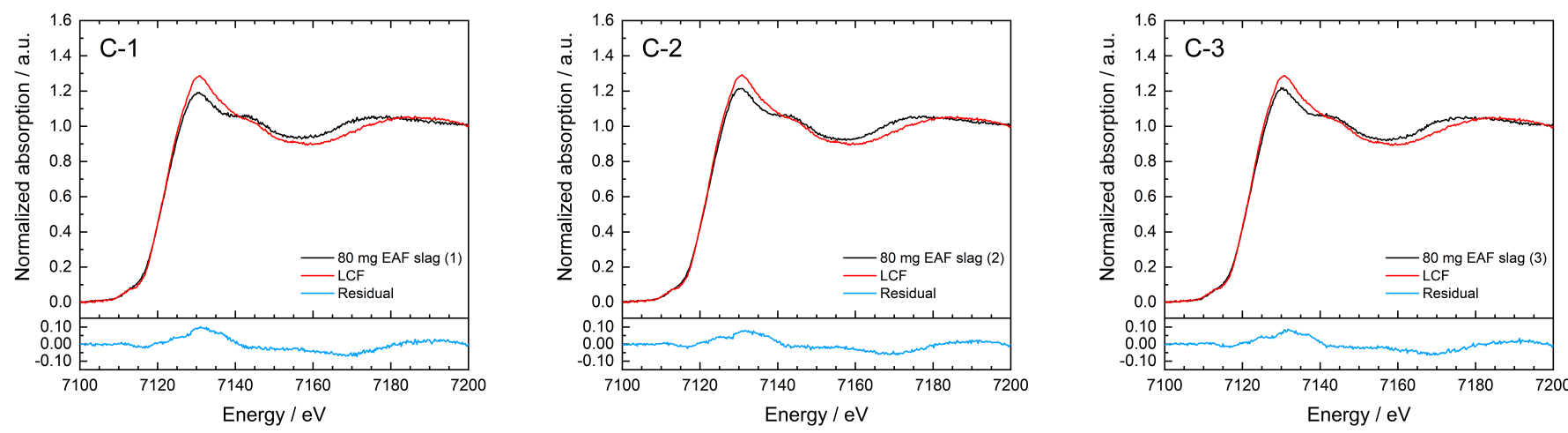

Figure S10: Recorded spectra of the three 80 mg specimens with fit and residuals from the LCFs. These fits included magnetite, olivine and iron chromite as reference materials.

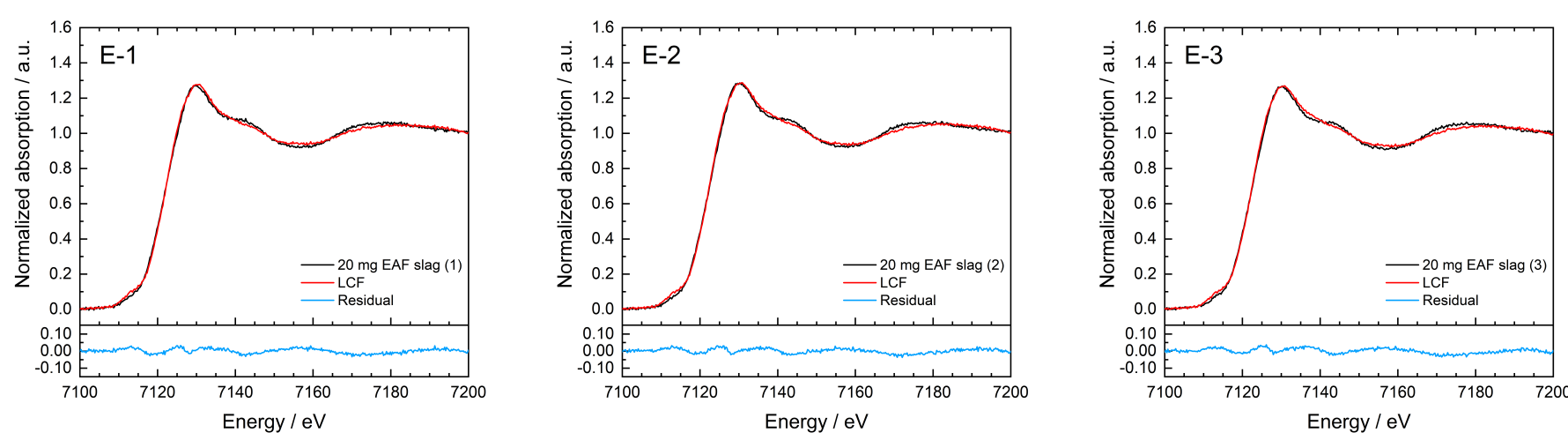

Figure S11: Recorded spectra of the three 20 mg specimens with fit and residuals from the mathematically best LCF. These fits included magnetite, olivine and iron chromite, haematite and metallic iron as reference materials.

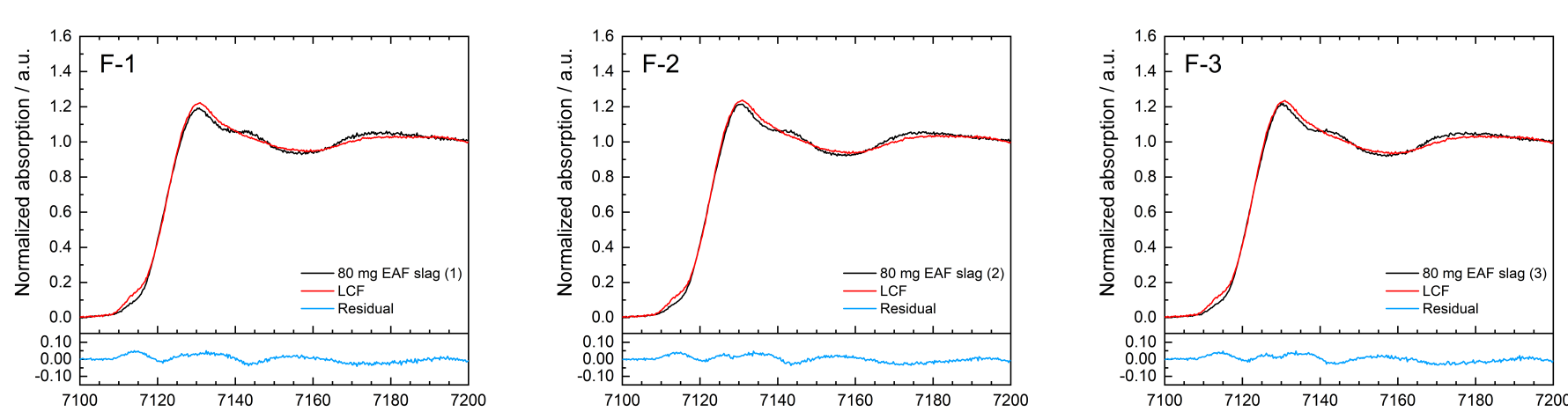

Figure S12: Recorded spectra of the three 80 mg specimens with fit and residuals from the mathematically best LCF. These fits included magnetite, olivine and iron chromite, haematite and metallic iron as reference materials.

Figure S13 shows the recorded XANES spectra of the reference materials that were used for the speciation analysis of the EAF slag through linear combination fitting.

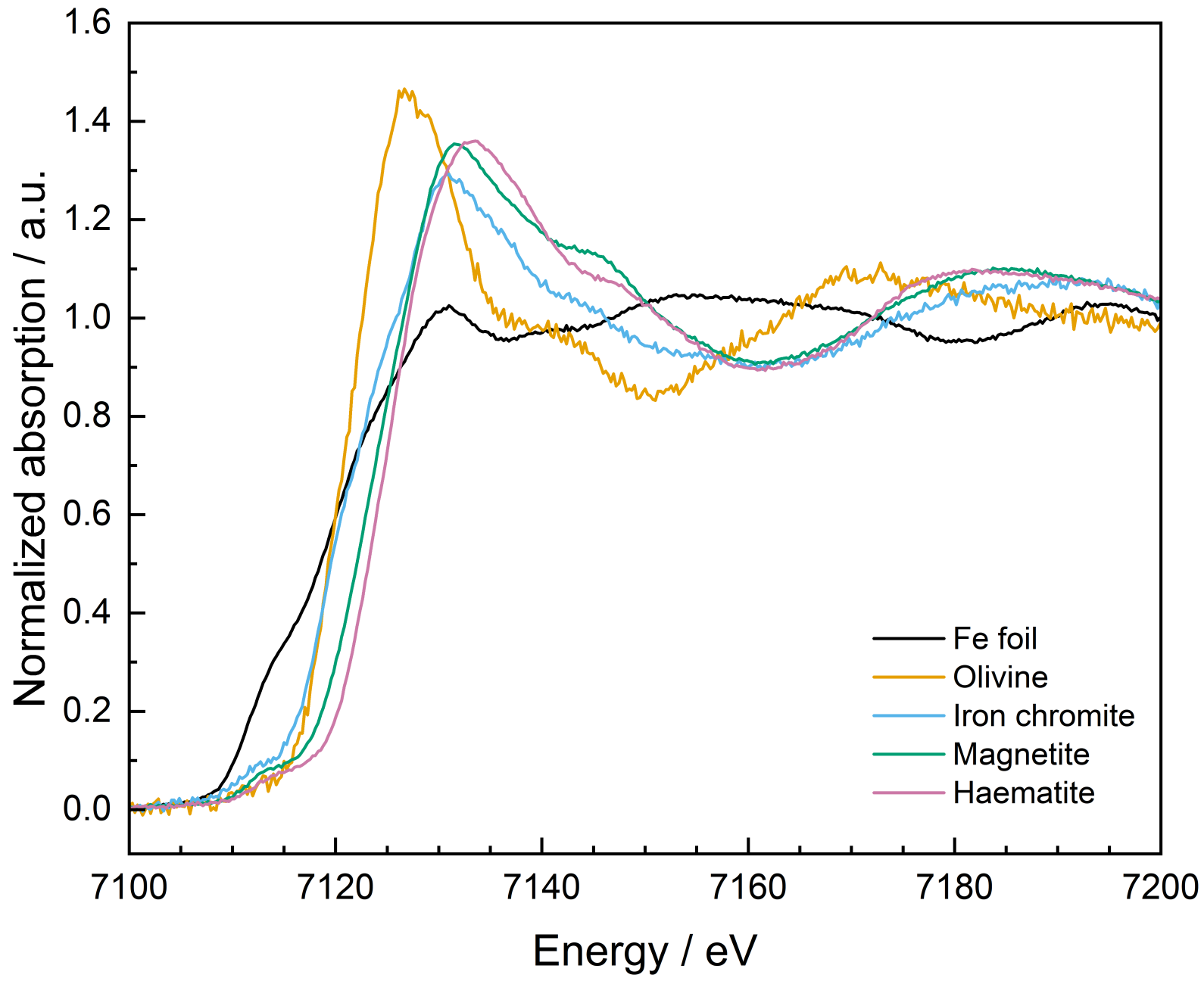

Figure S13: XANES spectra of reference materials used for speciation analysis through linear combination fitting.

References

(1) Hampel, S.; Alhafez, I. A.; Schnickmann, A.; Wunderlich, S.; Li, H.; Fischlschweiger, M.; Schirmer, T.; Merkert, N.; Fittschen, U. E. A. Experimental and Simulation Studies on the Mn Oxidation State Evolution of a Li2O-MnOx-CaO-SiO2 Slag Analogue. *Minerals* **2024**, *14*.

(2) Riesemeier, H.; Ecker, K.; Görner, W.; Müller, B.; Radtke, M.; Krumrey, M. Layout and first XRF applications of the BAMline at BESSY II. *X-Ray Spectrometry* **2005**, *34*, 160–163.

(3) Ravel, B.; Newville, M. ATHENA, ARTEMIS, HEPHAESTUS: data analysis for X-ray absorption spectroscopy using IFEFFIT. *J. Synchrotron Radiat.* **2005**, *12*, 537–541.

(4) Wittkowski, A.; Schirmer, T.; Qiu, H.; Goldmann, D.; Fittschen, U. E. A. Speciation of Manganese in a Synthetic Recycling Slag Relevant for Lithium Recycling from Lithium-Ion Batteries. *Metals* **2021**, *11*, 188.

(5) McMaster, W. H., Del Kerr Grande, N., Mallett, J. H., Hubbell, J. H., Eds. *Compilation of X-Ray Cross Sections: Lawrence Livermore National Laboratory Report UCRL-50174 Sec. II Rev. 1*; Lawrence Radiation Laboratory, University of California: Livermore, 1969.

(6) Gates-Rector, S.; Blanton, T. The Powder Diffraction File: a quality materials characterization database. *Powder Diffraction* **2019**, *34*, 352–360.
